# Exploration of chemical components and metabolite synthesis pathways in eight *Ephedra* species based on HS-GC-MS and UPLC-Q-TOF-MS

**DOI:** 10.3389/fpls.2024.1421008

**Published:** 2024-06-12

**Authors:** Bing Guo, Lina Yang, Hengyang Li, Qi An, Yongli Liu, Jie Cheng, Fangjie Hou, Long Guo, Dan Zhang

**Affiliations:** ^1^ Traditional Chinese Medicine Processing Technology Innovation Centre of Hebei Province, College of Pharmacy, Hebei University of Chinese Medicine, Shijiazhuang, China; ^2^ International Joint Research Centre on Resource Utilization and Quality Evaluation of Traditional Chinese Medicine of Hebei Province, College of Pharmacy, Hebei University of Chinese Medicine, Shijiazhuang, China; ^3^ Department of Chinese Materia Medica, Hebei Institute for Drug and Medical Device Control, Shijiazhuang, China; ^4^ Hebei Industrial Technology Institute for Traditional Chinese Medicine Preparation, The First Affiliated Hospital of Hebei University of Chinese Medicine, Shijiazhuang, China

**Keywords:** *Ephedra species*, chemical component, molecular network, synthetic route, HS-GC-MS, UPLC-Q-ToF-MS

## Abstract

**Objective:**

*Ephedra*, widely used in clinical practice as a medicinal herb, belongs to the genus *Ephedra* in the family Ephedraceae. However, the presence of numerous *Ephedra* varieties and variants requires differentiation for accurate identification.

**Methods:**

In this study, we employed headspace gas chromatography mass spectrometry (HS-GC-MS), ultra-high performance liquid chromatography coupled with quadrupole time-of-flight mass spectrometry (UPLC-Q-TOF-MS), and global natural products social molecular networking (GNPS) for chemical component identification. Chemometric analysis was used to analyze the differential components. Metabolic analysis and Kyoto encyclopedia of genes and genomes (KEGG) enrichment were utilized to explore the synthesis pathways of different components.

**Result:**

A total of 83 volatile and 79 non-volatile components were identified in *Ephedra* species. Differential analysis revealed that among the eight *Ephedra* stems, 18 volatile and 19 non-volatile differential compounds were discovered, whereas *Ephedra* roots exhibited 21 volatile and 17 non-volatile markers. Volatile compounds were enriched in four synthetic pathways, while non-volatile components were enriched in five pathways among the differentiated components.

**Conclusion:**

This study is the first to conduct a comparative analysis of chemical components in different *Ephedra* species and parts. It provides a foundational reference for authenticating *Ephedra* herbs, evaluating medicinal resources, and comparing quality in future studies.

## 1 Introduction

The *Ephedra* genus (Ephedraceae) comprises 69 species, four subspecies, and two accepted varieties, all widely distributed in arid and semi-arid regions of Asia, Europe, Northern Africa (Sahara), southwestern North America and South America (González-Juárez et al., 2020). Traditionally, *Ephedra* species have been employed to alleviate various ailments such as allergies, bronchial asthma, chills, colds, coughs, edema, fever, flu, headaches, and nasal congestion (Abourashed et al., 2003). In China, there are 12 species and 4 varieties of *Ephedra* distributed throughout the country, except in provinces and regions in the lower reaches of the Yangtze River and the Pearl River Basin. These species are more abundant in the northwest provinces and regions, as well as in the high mountain areas of Yunnan and Sichuan. Records indicate the following species: *Ephedra praewalskii* Stapf, *E. intermedia* Schrenk ex Mey, *E. sinica* Stapf, *E. equisetina* Bunge, *E. saxatilis* Royle ex Florin, *E. likiangensis* Florin, *E. lepidosperma* C. Y. Cheng, *E. minuta* Florin, *E. gerardiana* Wall, *E. monosperma* Gmel. ex Mey, *E. regeliana* Florin, and *E. fedtschenkoae* Pauls (Editorial Committee of Flora of China, C. A. o. S, 1979), et al.

*E. sinica* Stapf, known as “Mahuang” in China, has been utilized as a stimulant and antiasthmatic for over 5,000 years and continues to be employed in *Ephedra* preparations and extracts worldwide. The 2020 edition of the Pharmacopoeia of the People’s Republic of China categorizes Ephedrae herba as the dried straw stems of *E. sinica* Stapf, *E. intermedia* Schrenk et C. A. Mey., or *E. equisetina* Bge. Ephedrae radix et rhizoma comprise the roots and rhizome of *E. sinica* Stapf, *E. intermedia* Schrenk et C. A. Mey (Commission, 2020). To date, approximately 300 components spanning eight categories (alkaloids, volatile oils, flavonoids, polysaccharides, simple phenylpropanoids, condensed tannins, organic acids, and sterols) have been identified from the three legally recognized species of *Ephedra* (*E. sinica*, *E. intermedia*, *E. equisetina*). Despite originating from the same plant, Ephedrae herba and Ephedrae radix et rhizoma contain distinct types of alkaloids, resulting in differences in clinical applications (Tian et al., 2022). Phenylpropanoid alkaloids constitute the alkaloid component of *Ephedra* stem, whereas macrocyclic spermine alkaloids are found in *Ephedra* root.

The chemical components of *Ephedra* species vary not only between its medicinal parts but also among different species, leading to variations in the types and contents of chemical components (Sun et al., 2018). However, the chemical components and their corresponding pharmacological effects across different species and parts of *Ephedra* remain unexplored. Further comprehensive investigations are warranted, especially regarding the chemical compositions of *Ephedra* species in Chinese ethnomedicine, which could potentially serve as a resource for alternative medicinal species. Given the frequent use of *Ephedra* as a medicinal plant in China and worldwide, it is imperative to enhance our understanding of its traditional uses and chemical characteristics. Previous studies have compared different species of *Ephedra*, such as *E. monosperma* Gmel. ex Mey, *E. alata*, and *E. gerardiana* Wall, in terms of their total alkaloids, phenolic acids, and flavonoids content. It was observed that *E. alata* exhibited higher levels of phenolic acids and flavonoids (Ibragic and Sofić, 2015) These findings underscore significant differences in the chemical compositions of various *Ephedra* species, highlighting the importance of comparing and identifying *Ephedra* species based on their chemical constituents. Geographically, it has been observed that the total alkaloid content of *E. sinica* and *E. equisetina* in eastern and central Mongolia is 1.43% higher than that in *Ephedra* from other regions (Kitani et al., 2009). Additionally, ephedrine levels increase with altitude, while pseudoephedrine levels decrease with altitude (Lu et al., 2023). Thus, variations in the contents and proportions of primary and secondary metabolites among different regions and species of *Ephedra* reflect differences in their metabolic activities (Loera et al., 2012).

In this study, eight common plants from the *Ephedra* genus in China, namely, *E. sinica* Stapf, *E. intermedia* Schrenk et C. A. Mey, *E. equisetina* Bge., *E. gerardiana* Wall, *E. likiangensis* Florin, *E. przewalskii* Stapf, *E. saxatilis* Royle ex Florin, *E. monosperma* Gmel. Ex Mey., were selected for chemical composition evaluation. HS-GC-MS, UPLC-Q-TOF-MS combined with GNPS technology were used to analyze extracted samples from various parts of *Ephedra* species, acquire metabolite information, and elucidate the similarities and differences in chemical compositions among these *Ephedra* plants. Finally, KEGG enrichment analysis was conducted to investigate differential components and their associated synthetic pathways. Based on these findings, the differences and similarities in metabolite profiles among the eight *Ephedra* species were analyzed and compared, thereby establishing a foundation for the comprehensive utilization and further research of *Ephedra* plants.

## 2 Materials and methods

### 2.1 Reagents and materials

Methanol, acetonitrile, and formic acid of LC-MS grade were obtained from Fisher Scientific (Pittsburgh, PA, USA), while ultrapure water was generated using a synergy water purification system (Millipore, Billerica, MA, USA). The internal standard hyperoside (≥ 98.0%, Lot no. Y20A9X59340) was procured from Chengdu Push-Biotechnology Co. Ltd. (Chengdu, China). All other chemicals and reagents were of analytical grade.

*E. sinica* Stapf, *E. intermedia* Schrenk et C. A. Mey, *E. equisetina* Bge., *E. gerardiana* Wall, *E. likiangensis* Florin, *E. przewalskii* Stapf, *E. saxatilis* Royle ex Florin, *E. monosperma* Gmel. Ex Mey. were collected from different regions of China and authenticated by Professor Dan Zhang of Hebei University of Chinese Medicine. Detailed information of each sample can be found in **Table 1** and **Supplementary Figure S1**. Specimens were stored at Hebei University of Chinese Medicine (Shijiazhuang, Hebei, China).

**Table 1:** Sample information of MHS and MHR.

Part	Sample	Source	Collection area
Stem (MHS)	SX	*Ephedra sinica* Stapf	Yuzai County, Shanxi Province
	NMG	*E. sinica* Stapf	Neimenggu Autonomous Region
	WQC	*E. sinica* Stapf	Wanquan District, Hebei Province
	GS	*E. intermedia* Schrenk et C. A. Mey	Gansu Province
	WQZ	*E. intermedia* Schrenk et C. A. Mey	Wanquan District, Hebei Province
	QYZ	*E. equisetina* Bge.	Wuan District, Hebei Province
	WQM	*E. equisetina* Bge.	Wanquan District, Hebei Province
	SL	*E. gerardiana* WALL	Yunnan Province
	LJ	*E. likiangensis* Florin	Lijiang City, Yunnan Province
	MG	*E. przewalskii* Stapf	Xinjiang Autonomous Region
	XZ	*E. saxatilis* Royle ex Florin	Xizang Autonomous Region
	WQD	*E. monosperma* Gmel. Ex Mey.	Wanquan District, Hebei Province
Root (MHR)	WQC	*E. sinica* Stapf	Wanquan District, Hebei Province
	WQZ	*E. intermedi*a Schrenk et C. A. Mey	Wanquan District, Hebei Province
	GS	*E. intermedia* Schrenk et C. A. Mey	Gansu Province
	NMG	*E. equisetina* Bge.	Wanquan District, Hebei Province
	MG	*E. przewalskii* Stapf	Xinjiang Autonomous Region
	XZ	*E. saxatilis* Royle ex Florin	Xizang Autonomous Region
	SL	*E. gerardiana* Wall	Yunnan Province
	LJ	*E. likiangensis* Florin	Lijiang City, Yunnan Province
	WQD	*E. monosperma* Gmel. Ex Mey.	Wanquan District, Hebei Province

### 2.2 Sample preparation

HS-GC-MS sample preparation involved crushing the *Ephedra* samples to a uniform size (sieved through a 20 mesh sieve) and sealing them at room temperature before further experiments. Each *Ephedra* plant powder (1.5 g) was accurately weighed and sealed in a 10 mL headspace bottle. Equal amounts of each sample were thoroughly mixed to prepare a quality control (QC) sample. The QC sample was inserted every five samples to ensure the stability, repeatability, and reproducibility of the GC-MS method.

For UPLC-Q-TOF-MS sample preparation, 0.5 g of each *Ephedra* powder was accurately weighed and placed in 65% methanol (v/v) comprising 15 mL of methanol. The mixture underwent sonication for 30 minutes to achieve appropriate dilution, followed by centrifugation at 13,000 rpm for 10 minutes. For the positive ionic mode, concentrations of 3.3 mg/mL and 6.6 mg/mL were utilized for MHS and MHR, respectively, with an internal standard content of 0.01 mg/mL. For the negative ionic mode, a concentration of 16.5 mg/mL was utilized for both stem and root of *Ephedra*, with an internal standard content of 0.04 mg/mL. The resulting solution was filtered through a 0.22 μm microporous membrane. To evaluate the LC–MS reproducibility during analysis, 100 μL of each sample solution was thoroughly mixed well to prepare a QC sample. QC samples were inserted in every five sample to ensure the stability, repeatability, and reproducibility of the LC–MS method.

### 2.3 Analysis of volatile components of *Ephedra* based on HS-GC-MS

GC-MS analysis was conducted using an Agilent 7890–5977B gas chromatography-mass spectrometry system (Agilent Technologies, Santa Clara, CA, USA) with an HP-5MS fused silica capillary column (30 m × 0.25 mm, 0.25 µm film thickness; Agilent Technologies, Santa Clara, CA, USA) utilizing an electron impact (EI) ionization chamber and operating in full scan mode.

Headspace injection was carried out on an Agilent 7697A autosampler (Agilent Technologies, Santa Clara, CA, USA) with 10 mL HS vials. The headspace operating conditions were as follows: sample equilibration temperature, 120°C; sample loop temperature, 130°C; transfer line temperature, 140°C; sample bottle pressurization pressure, 15 psi; vial pressurization time, 12 s; sample loop fill time, 12 s; and transfer time, 20 s. Prior to GC analysis, the sample vial (20 mL) was vigorously shaken for 15 min during equilibration.

For the MHS protocol, the injector temperature was set to 250°C in a split mode (20:1), and the carrier gas used was helium (99.999% pure) with a flow rate of 1.0 ml/min. The initial temperature was maintained at 60°C for 5 minutes, then raised to 80°C at a rate of 1°C/min, where it was held for 5 minutes. Subsequently, a ramp to 200°C was initiated at a rate of 10°C/min, which was maintained for 2 minutes, resulting in a total analysis time of 44 minutes.

For the MHR protocol, the injector temperature was set to 250°C in a split mode (20:1), and the carrier gas used was helium (99.999% pure) with a flow rate of 1.0 ml/min. The initial temperature was set to 60°C for 7 minutes, then increased to 75°C at a rate of 1°C/min, where it was held for 2 minutes. Subsequently, a ramp to 180°C was initiated at a rate of 5°C/min, which was maintained for 2 minutes, resulting in a total analysis time of 47 minutes.

For MS conditions, the EI source temperature was set to 230°C, quadrupole temperature to 150°C, and electron energy to 70 eV. The solvent delay time was 3 minutes, and the mass scan range was from *m/z* 50 to 500 in the full scan mode.

Total ion flow chromatograms of *Ephedra* obtained via GC-MS were analyzed to acquire mass spectra of chromatographic peaks. Data were analyzed and processed using Agilent MassHunter Qualitative Analysis Navigator software (version B.08.00, Agilent Technologies, Inc., Santa Clara, CA, USA), with the peak filter set to a relative peak area of 5000. The mass spectral data of the measured components were compared with the National Institute of Standards and Technology (NIST) 17.0 L standard mass spectral search database (match >80%) to determine the chemical components, and volatile components in the samples were qualitatively analyzed. Relative quantification of the components was conducted using Agilent MassHunter Quantitative Analysis software (version B.09.00, Agilent Technologies, Inc., Santa Clara, CA, USA) based on peak area normalization method.

### 2.4 Analysis of non-volatile components of *Ephedra* based on UPLC-Q-TOF-MS with molecular network

UPLC-MS analysis was conducted using an Agilent 1290 Infinity II system coupled with an Agilent 6545 quadrupole time-of-flight mass spectrometer (Q-TOF-MS) system equipped with an electrospray ionization interface (Agilent Technologies, Santa Clara, CA, USA).

Sample separation was performed using a Waters Acquity UPLC HSS T3 column (2.1 × 100 mm, 1.8 μm) with a flow rate of 0.3 mL/min and a column temperature of 30°C. The binary gradient elution system comprised acetonitrile (B) and water with 0.1% formic acid (A). The gradient elution protocol for MHS and MHR was optimized as follows: 0–3 min, 5% B; 3–6 min, 5–10% B; 6–20 min, 10–14% B; 20–23 min, 14–20% B; 23–25 min, 20–30% B; 25–28 min, 30–40% B; 28–30 min, 44–55% B; 30–40 min, 55–85% B. The injection volume was set to 1.0 µL.

The MS acquisition parameters were configured as follows: drying gas (N_2_) temperature, 320°C; sheath gas temperature, 350°C; drying gas (N_2_) flow rate, 10.0 L/min; sheath gas flow (N_2_) rate, 11 L/min; nebulizer gas pressure, 35 psi; capillary voltage, 3500 V; fragmentor voltage, 135 V; collision energy, 40 eV. The analysis was conducted in both positive and negative modes with a mass range of *m/z* 50–1000 Da. Data was analyzed using Agilent MassHunter Qualitative Analysis Software (version B.10.00, Agilent Technologies, Palo Alto, CA, USA). All components were identified utilizing MS data and MS/MS fragment patterns from the MN chemical composition database, TCMSP (http://lsp.nwu.edu.cn/tcmsp.php), MassBank (https://massbank.eu/MassBank/Search), Agilent herbal library-v20–04-17, Chemspider (http://www.chemspider.com/), secondary mass spectrometry debris ion speculation, and published literature.

The data, which included molecular ion peak mass-to-charge ratios for determining possible molecular formulas of components, were analyzed using Agilent MassHunter qualitative software (version B.10.00, Agilent Technologies, Palo Alto, CA, USA). Additionally, molecular networking (MN) was constructed from UPLC-MS/MS data of MHS and MHR. All MS/MS data files were converted to 32-bit mzXML format using MSconvert software and subsequently uploaded to the GNPS platform (https://gnps.ucsd.edu) via WinSCP (https://winscp.net). The MN was then generated according to the online workflow (https://ccms-ucsd.github.io/GNPSDocumentation/quickstart) with specific parameters: minimum cosine value of 0.70, minimum matching peak of 6, parent mass and fragmentation tolerance of 0.02 Da, maximum connected component size of 100, and minimum cluster size of 1. MScluster was not executed. Finally, the results were exported to Cytoscape 3.9.1 software for visualization.

### 2.5 MetaboAnalyst and kyoto encyclopedia of genes and genomes enrichment analysis

Differential components were subjected to pathway enrichment analysis, annotation, visualization, and integrated discovery using the MetaboAnalyst database (MetaboAnalyst) (https://www.metaboanalyst.ca/), with the “Chemical structures” option selected. This process generated an automatic visualization bubble diagram, where dot color corresponds to different p-values, and dot size indicates expression levels in enriched types. Additionally, the synthesis pathways of the identified differential compounds were explored using relevant literature and the KEGG database (https://www.genome.jp/kegg/compound/) (Li et al., 2023).

### 2.6 Data analytics

The data were analyzed using IBM SPSS Statistics 23.0 for statistical analysis and presented as mean ± SD. Chemical structure formulae and synthetic pathway diagrams were generated using ChemDraw 20.0 software, while graphs were generated and analyzed with GraphPad Prism 9 and Origin 2021 software. Principal component analysis (PCA), Partial Least Squares-Discrimination Analysis (PLS-DA), and Orthogonal Partial Least Squares-Discriminant Analysis (OPLS-DA) models were performed using Simca-p 14.1 software (SIMCA Imola s. c., Imola, Bologna, Italy).

## 3 Results

### 3.1 Comparison of volatile components of eight *Ephedra* species using HS-GC-MS

#### 3.1.1 Comparison of volatile component types and their relative contents

**Figure 1:**
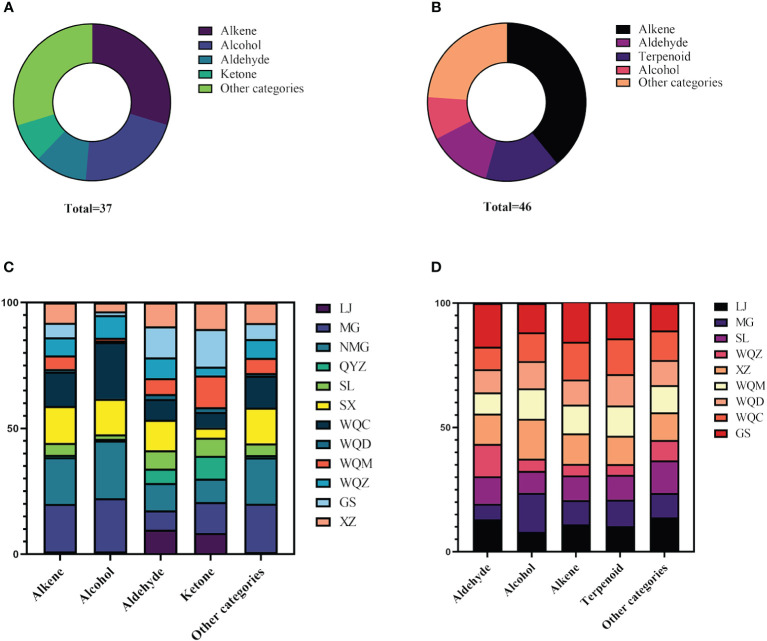
Distribution of different categories of volatile components in MHS **(A)** and MHR **(B)**, and comparison of volatile components in MHS **(C)** and MHR **(D)** from eight *Ephedra* species.

**Figure 2:**
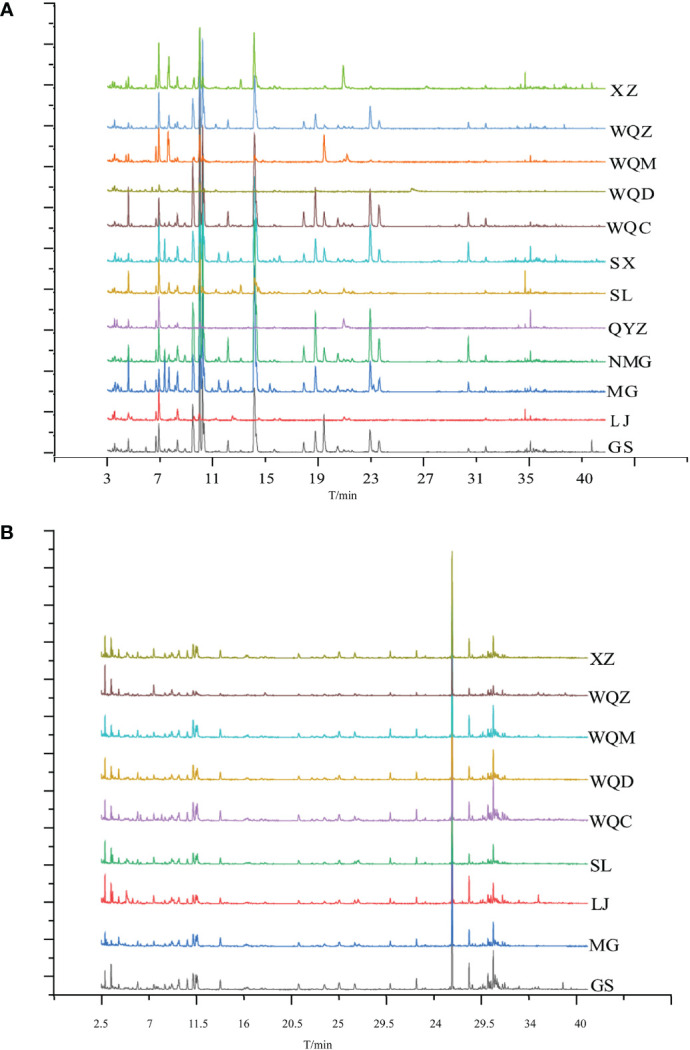
Total ion flow diagrams of MHS **(A)** and MHR **(B)** for various species.

Relative to the MHS of eight *Ephedra* species, MG, NMG, SX, and WQC exhibited higher levels of alkenes and alcohols. LJ, NMG, SX, and GS showed higher levels of aldehydes, while MG, WQZ, GS, and XZ showed higher levels of ketones. Among the MHR, LJ, GS, and WQZ exhibited higher relative content of aldehydes, while MG and XZ showed higher content of alcohols. Additionally, LJ, XZ, and GS exhibited higher levels of alkenes. WQC and WQZ had lower contents of alcohols, aldehydes, and terpenoids. Detailed information on the relative percentage content of each volatile component of MHS and MHR is presented in **Table 2** and **Table 3**, as illustrated in **Figure 1** and **Figure 2**.

**Table 2:** Relative percentage content of each volatile component in MHS.

No.	*t_R_* (min)	Metabolites	CAS number	Class	WQM(%)	QYZ(%)	XZ(%)	WQD(%)	SX(%)	SL(%)	NMG(%)	WQC(%)	LJ(%)	GS(%)	MG(%)	WQZ(%)
1	3.517	Furfural	98–01-1	Aldehyde	8.50	3.80	16.28	4.39	3.29	11.43	5.09	3.65	7.49	23.84	7.43	4.81
2	3.677	1,6-Dimethylhepta-1,3,5-triene	139705–56-9	Alkene	9.16	10.67	7.30	7.23	7.66	6.48	6.51	7.31	10.07	7.46	8.80	11.36
3	4.215	1-Pentanol, 3-methyl-	589–35-5	Alcohol	4.22	5.74	9.96	8.41	10.51	6.62	14.00	6.39	7.78	9.64	11.07	5.66
4	4.618	Bicyclo[2.2.1]hept-2-ene,2,3-dimethyl-	529–16-8	Alkene	26.58	9.17	21.43	3.42	3.66	6.27	4.06	3.29	6.60	5.42	4.73	5.36
5	4.808	Styrene	100–42-5	Alkene	5.24	4.66	6.89	0.05	5.27	14.86	11.72	18.14	2.46	1.88	25.65	3.16
6	5.086	Heptanal	111–71-7	Aldehyde	3.61	4.26	6.47	6.27	4.77	5.61	7.69	6.47	27.10	14.97	6.42	6.36
7	7.080	2-Heptanone,6-methyl-	928–68-7	Ketone	15.11	7.32	11.65	2.11	3.41	6.61	10.49	5.95	6.75	12.82	15.01	2.78
8	7.323	Benzaldehyde	100–52-7	Aldehyde	7.81	7.37	9.92	1.78	11.16	8.82	7.92	6.22	10.37	13.72	6.22	8.69
9	7.796	2*H*-Pyran,2-ethenyltetrahydro-2,6,6-trimethyl-	7392–19-0	Pyan	1.49	0.00	3.34	0.00	30.68	2.79	14.45	5.23	0.00	0.17	39.75	2.10
10	8.147	2-Methyl-1-octen-3-yne	17603–76-8	Alkyne	21.53	1.83	18.84	1.50	2.82	6.87	3.90	2.75	0.61	21.80	9.38	8.17
11	8.663	5-Hepten-2-one,6-methyl-	110–93-0	Ketone	6.92	4.45	27.49	4.30	7.09	4.96	7.64	7.61	5.92	4.37	10.42	8.83
12	8.836	Furan,2-pentyl-	3777–69-3	Pyan	3.78	3.73	10.59	3.75	11.68	7.08	12.61	10.46	9.11	8.77	11.90	6.56
13	9.473	*α*-Phellandrene	99–83-2	Alkene	2.76	2.10	6.92	1.25	12.92	5.97	20.51	14.39	1.71	3.61	16.28	11.58
14	10.119	7-Oxabicyclo[2.2.1]heptane, 1-methyl-4-(1-methylethyl)-	470–67-7	Alcohol	0.80	0.07	0.80	0.09	11.18	0.51	21.50	30.80	0.01	0.19	24.17	9.89
15	10.679	*O*-Cymene	527–84-4	Alkene	2.00	0.61	4.55	0.77	16.46	3.89	25.34	13.36	0.56	1.79	22.92	7.76
16	10.917	*D*-Limonene	5989–27-5	Akene	0.67	0.13	1.01	0.15	15.63	0.71	27.35	19.01	0.20	0.47	21.05	13.62
17	11.047	Eucalyptol	470–82-6	Phenolic	1.74	0.82	1.56	2.24	16.87	2.26	17.64	27.35	0.41	2.04	16.85	10.22
18	12.265	Furan,tetrahydro-2,2-dimethyl-5-(1-methyl-1-propenyl)-	7416–35-5	Pyan	2.26	0.00	11.15	0.00	24.62	3.97	12.85	6.35	0.00	1.68	35.27	1.86
19	12.994	*γ*-Terpinene	99–85-4	Alkene	0.42	0.01	7.73	0.10	10.74	2.26	24.91	21.27	0.02	0.62	21.43	10.47
20	14.043	Ethyl 2-(5-methyl-5-vinyltetrahydrofuran-2-yl) propan-2-ylcarbonate	1000373–80-32	Pyan	2.32	0.42	26.27	0.00	10.54	23.69	10.63	5.21	1.39	2.61	14.69	2.23
21	15.369	3,5-Dimethylanisole	874–63-5	Ether	0.12	0.00	2.14	0.00	29.17	4.31	22.58	17.40	0.00	0.00	15.76	8.53
22	15.365	*P*-(1-Propenyl)-toluene	1000429–54-9	Alkene	0.57	0.00	2.34	0.00	12.59	2.88	26.86	20.48	0.01	0.16	24.06	10.04
23	16.839	3,4-Dimethylcyclohexanol	5715–23-1	Alcohol	4.85	4.79	14.01	6.28	8.26	6.91	8.94	7.71	6.85	11.75	11.49	8.16
24	17.229	(2*S*,4*R*)-4-Methyl-2-(2-methylprop-1-en-1-yl)tetrahydro-2*H*-pyran	3033–23-6	Pyan	1.99	2.73	9.68	6.87	34.23	4.53	2.21	3.00	15.54	17.49	1.49	0.25
25	19.289	3-Cyclohexen-1-ol,1-methyl-4-(1-methylethyl)-	586–82-3	Alcohol	0.63	0.00	0.44	0.00	11.87	0.59	22.65	29.06	0.09	0.18	24.10	10.39
26	20.221	Cyclohexanol,1-methyl-4-(1-methylethenyl)-	138–87-4	Alcohol	0.44	0.00	0.17	0.00	12.58	0.22	27.74	27.70	0.05	0.11	23.71	7.27
27	20.984	Benzene,1-ethenyl-4-methoxy-	637–69-4	Alkene	35.43	1.47	3.85	0.00	15.58	1.68	13.26	14.75	0.00	4.48	4.74	4.75
28	22.531	1,2-Propanedione,1-phenyl	579–07-7	Ketone	4.63	18.09	0.08	0.00	5.46	10.37	4.84	6.98	16.23	27.30	1.80	4.21
29	23.208	3-Cyclohexen-1-ol,4-Methyl-1-(1-methylethyl)-,(*R*)-	20126–76-5	Alcohol	1.32	0.98	35.70	1.61	9.68	2.88	13.54	11.88	0.58	1.46	13.34	7.03
30	24.734	*α*-Terpineol	98–55-5	Alcohol	0.61	0.00	0.57	0.12	17.36	0.96	24.73	22.26	0.26	0.33	22.38	10.41
31	25.484	*α*-Terpinyl acetate	80–26-2	Salts	0.52	0.00	2.32	0.00	13.54	0.16	24.98	28.59	0.05	0.07	23.00	6.76
32	32.772	1-Cyclohexene-1-carboxaldehyde,4-(1-methylethyl)-	21391–98-0	Aldehyde	0.91	0.00	6.53	0.00	22.86	0.13	27.36	19.23	0.00	0.28	14.21	8.50
33	34.146	Cyclohexanemethanol, 4-hydroxy-α,α,4-trimethyl-	80–53-5	Alcohol	5.39	5.31	2.18	2.88	10.97	7.65	14.09	16.19	6.67	6.19	12.71	9.77
34	36.751	Octane, 6-ethyl-2-methyl-	62016–19-7	Alkane	6.53	7.87	15.59	4.41	8.11	7.94	7.67	7.52	8.98	8.18	7.13	10.07
35	37.415	*α*-Bisabolol	515–69-5	Alcohol	2.57	5.06	6.48	0.77	8.41	20.84	7.17	3.08	14.48	20.90	6.85	3.39
36	37.419	*Cis*-*α*-Bergamotene	18252–46-5	Alkene	2.39	4.44	8.16	0.67	6.03	21.95	5.44	3.24	14.82	23.08	7.08	2.71
37	37.857	Alloaromadendrene	25246–27-9	Alkene	7.11	17.95	9.04	0.78	17.43	5.14	11.26	8.75	8.02	4.53	5.40	4.58

**Table 3:** Relative percentage content of each volatile component in MHR.

No.	*t_R_* (min)	Metabolites	CAS number	Class	LJ(%)	MG(%)	SL(%)	WQZ(%)	XZ(%)	WQM(%)	WQD(%)	WQC(%)	GS(%)
1	2.921	Hexanal	66–25-1	Aldehyde	14.33	8.48	10.46	14.83	10.93	9.79	11.34	9.89	9.94
2	3.021	2,3-Butanediol	513–85-9	Alcohol	4.87	30.99	9.11	1.77	23.68	11.43	4.81	8.53	4.81
3	3.485	Furfural	98–01-1	Aldehyde	12.42	4.37	10.28	11.00	13.67	7.36	10.71	8.41	21.77
4	3.615	Cyclopentanecarboxylic acid,1-methyl-	5217–05-0	Amide	36.13	0.49	23.87	2.62	10.93	8.05	8.23	4.40	5.28
5	4.178	1-Hexanol	111–27-3	Alcohol	9.88	13.09	9.94	10.97	13.08	10.27	15.77	8.49	8.51
6	4.889	4-Cyclopentene-1,3-diol,*cis*-	29783–26-4	Alkenol	33.98	0.46	21.50	4.38	13.81	6.68	8.89	4.14	6.16
7	5.045	Heptanal	111–71-7	Aldehyde	20.80	5.94	15.09	11.13	9.70	8.80	10.16	8.99	9.39
8	5.912	1,3,6-Heptatriene,2,5,6-trimethyl-	42123–66-0	Alkene	8.02	9.31	7.91	2.45	9.94	13.96	16.45	17.03	14.93
9	6.173	Bicyclo[3.1.1]hept-2-ene,3,6,6-trimethyl-	4889–83-2	Alkene	10.95	11.11	9.53	1.90	9.44	11.72	14.52	23.05	7.78
10	6.775	Camphene	79–92-5	Terpene	8.00	11.73	10.33	2.12	10.25	13.59	17.44	16.35	10.18
11	7.131	2-Heptanone, 6-methyl-	928–68-7	Ketone	7.87	9.91	10.03	23.58	18.93	8.27	7.38	6.08	7.94
12	7.378	Benzaldehyde	100–52-7	Aldehyde	12.39	9.38	11.94	15.00	13.63	9.87	8.02	9.67	10.10
13	7.651	2-Furancarboxaldehyde, 5-methyl-	620–02-0	Aldehyde	7.02	5.10	8.27	5.51	12.40	5.91	6.29	7.11	42.38
14	8.093	Bicyclo[3.1.1]heptane, 6,6-dimethyl-2-methylene-,(1*S*)-	18172–67-3	Alkene	23.23	3.42	9.79	1.07	6.56	8.49	5.69	38.71	3.03
15	8.405	1-Octen-3-ol	3391–86-4	Alkenol	8.52	9.03	7.88	8.56	15.57	13.26	9.82	10.34	17.03
16	8.799	5-Hepten-2-one,6-methyl-	110–93-0	Ketene	9.96	10.59	8.50	9.96	12.69	13.41	9.46	11.74	13.68
17	9.017	Furan,2-pentyl-	3777–69-3	Pran	12.69	12.58	10.26	12.39	9.75	11.25	8.82	10.61	11.65
18	9.216	Hexanoic acid	142–62-1	Acid	16.05	11.04	12.78	9.66	9.58	7.75	8.58	12.62	11.93
19	9.576	3,6-Heptadien-2-ol,2,5,5-trimethyl-,(*E*)-	26127–98-0	Alcohol	9.01	8.70	8.05	3.08	13.16	13.93	11.61	14.29	18.17
20	9.671	*α*-Phellandrene	99–83-2	Alkene	10.09	11.05	8.37	4.12	11.72	11.08	10.53	14.89	18.15
21	10.438	1,3-Cyclohexadiene,1-methyl-4-(1-methylethyl)-	99–86-5	Alkene	9.49	11.28	8.19	3.85	12.09	10.54	10.84	14.94	18.78
22	10.984	*O*-Cymene	527–84-4	Alkane	8.05	9.90	8.74	3.61	10.97	15.94	16.09	14.31	12.38
23	11.223	Cyclohexane,1-methylene-4-(1-methylethenyl)-	499–97-8	Alkene	10.09	10.92	8.98	3.77	11.67	11.90	11.39	14.93	16.35
24	11.353	Eucalyptol	470–82-6	Terpene	9.92	11.68	9.21	2.44	12.02	11.44	14.05	15.36	13.87
25	13.469	*γ*-Terpinene	99–85-4	Terpene	9.60	12.02	9.11	2.87	11.32	11.77	13.07	14.60	15.64
26	15.671	1,5-Heptadien-4-ol,3,3,6-trimethyl-	27644–04-8	Alkene	8.82	12.13	8.86	4.05	12.57	9.66	7.17	16.38	20.36
27	15.792	2,4,6-Octatriene,2,6-dimethyl-	673–84-7	Alkene	10.09	11.70	8.84	3.49	11.84	11.73	11.97	15.26	15.09
28	15.987	Benzene,(2-methyl-1-propenyl)-	768–49-0	Alkene	7.67	7.66	7.55	6.73	12.55	14.92	17.02	13.03	12.86
29	17.548	Nonanal	124–19-6	Aldehyde	11.30	4.97	9.61	20.90	13.15	9.42	9.39	9.94	11.31
30	20.609	(+)-2-Bornanone	464–49-3	Terpene	10.18	11.09	9.83	4.30	11.63	11.29	10.96	15.39	15.33
31	21.831	Cyclohexanone,5-methyl-2-(1-methylethyl)-,trans-	89–80-5	Ketone	12.04	6.05	8.51	11.74	10.00	14.49	18.33	11.13	7.69
32	22.967	Endo-Borneol	507–70-0	Cyclen ether terpene glycoside	9.20	10.57	9.23	5.20	11.88	10.41	9.29	13.47	20.76
33	24.302	Cyclohexanol,5-methyl-2-(1-methylethyl)-,(1*α*,2*β*,5*α*)-(.+/-.)-	15356–70-4	Alcohol	8.34	10.46	7.54	3.92	14.33	13.65	11.66	14.27	15.82
34	25.724	*α*-Terpineol	98–55-5	Alkenol	9.11	9.05	8.05	4.88	12.87	14.20	11.60	13.46	16.78
35	26.063	2-Methoxy-5-methylphenol	1195–09-1	Phenol	24.80	12.00	26.61	6.93	3.58	6.30	1.23	15.21	3.34
36	28.963	Benzene,1-methoxy-4-methyl-2-(1-methylethyl)-	31574–44-4	Aromatic ether	8.47	11.01	11.83	3.98	10.16	15.74	13.91	13.35	11.55
37	29.284	Carvone	99–49-0	terpene ketone	17.49	6.58	11.16	8.57	10.22	13.52	13.83	11.79	6.83
38	31.369	Bicyclo[2.2.1]heptan-2-ol,1,7,7-trimethyl-,acetate,(1*S*-endo)-	5655–61-8	Salts	8.89	11.21	10.55	5.08	12.39	12.37	9.83	14.06	15.61
39	32.167	Tridecane	629–50-5	Alkane	11.70	11.36	11.06	6.82	10.57	11.34	11.47	14.46	11.20
40	34.621	Ylangene	14912–44-8	Alkene	8.02	11.46	10.95	4.63	12.43	12.74	8.93	14.35	16.48
41	36.168	Caryophyllene	87–44-5	Ether	12.25	11.52	10.21	4.76	12.59	12.00	8.15	12.86	15.65
42	36.459	Benzene,1,4-dimethoxy-2-methyl-5-isopropyl-	14753–08-3	Alkene	5.45	12.16	10.03	4.84	14.74	9.60	7.08	14.77	21.32
43	37.417	(*E*)-*β*-Famesene	18794–84-8	Alkene	5.45	12.16	10.03	4.84	14.74	9.60	7.08	14.77	21.32
44	37.881	Naphthalene,1,2,4a,5,6,8a-hexahydro-4,7-dimethyl-1-(1-methylethyl)-	483–75-0	Alkene	7.41	11.30	10.15	5.75	14.11	10.47	6.50	12.97	21.33
45	38.375	(1*R*,3*aS*,4*aS*,8*aS*)-1,4,4,6-Tetramethyl-1,2,3,3a,4,4a,7,8-octahydrocyclopenta[1,4]cyclobuta[1,2]benzene	94535–52-1	Alkene	7.03	11.61	11.17	5.82	12.28	12.89	9.22	13.42	16.56
46	38.548	1*H*-Benzocycloheptene,2,4a,5,6,7,8-hexahydro-3,5,5,9-tetramethyl-,(*R*)-	1461–03-6	Alkene	6.12	12.29	12.94	3.31	14.21	11.25	6.17	10.98	22.73

#### 3.1.2 Chemometric analyses

**Figure 3:**
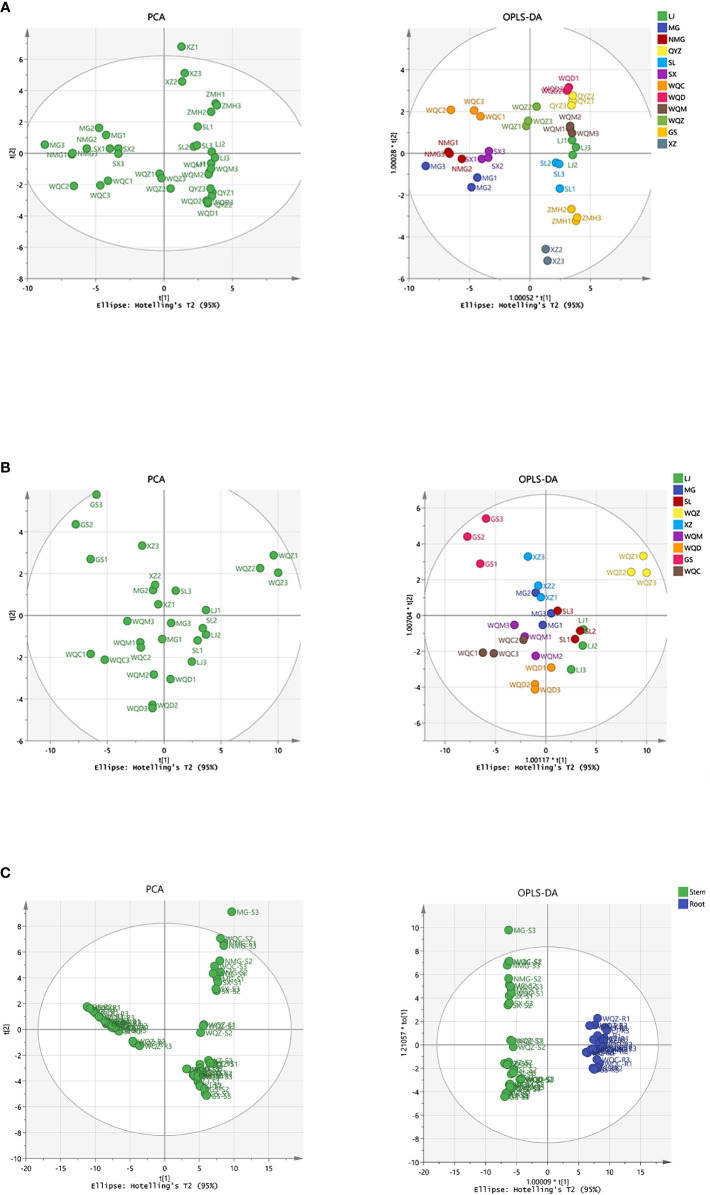
Multivariate statistical analysis of volatile components in MHS and MHR using PCA and OPLS-DA. **(A)** PCA and OPLS-DA with R^2^X, R^2^Y values of 0.970, 0.944 for MHS. **(B)** PCA and OPLS-DA with R^2^X, R^2^Y values of 0.887, 0.760 for MHR. **(C)** PCA and OPLS-DA with R^2^X, R^2^Y values of 0.784, 0.989 for MHS and MHR.

PCA was performed on the samples, revealing a clear separation between the varieties. Subsequently, a supervised OPLS-DA with a confidence interval of 95% was conducted based on PCA, demonstrating a significant separation between the varieties. The model exhibited robustness, with parameter indexes R^2^X > 0.5, R^2^Y > 0.5. The volatile compositional differences among *Ephedra* species were observed to be distributed across distinct regions, highlighting their notable variability. Furthermore, a comparison of volatile components between stems and roots indicated substantial disparities among these *Ephedra* species, as depicted in **Figure 3**. Eighteen distinct components were identified in 8 species of MHS, and twenty-one distinct components were identified in MHR, with VIP > 1 and *P* < 0.05, as shown in **Supplementary Table S1**.

#### 3.1.3 Analysis of differential components

**Figure 4:**
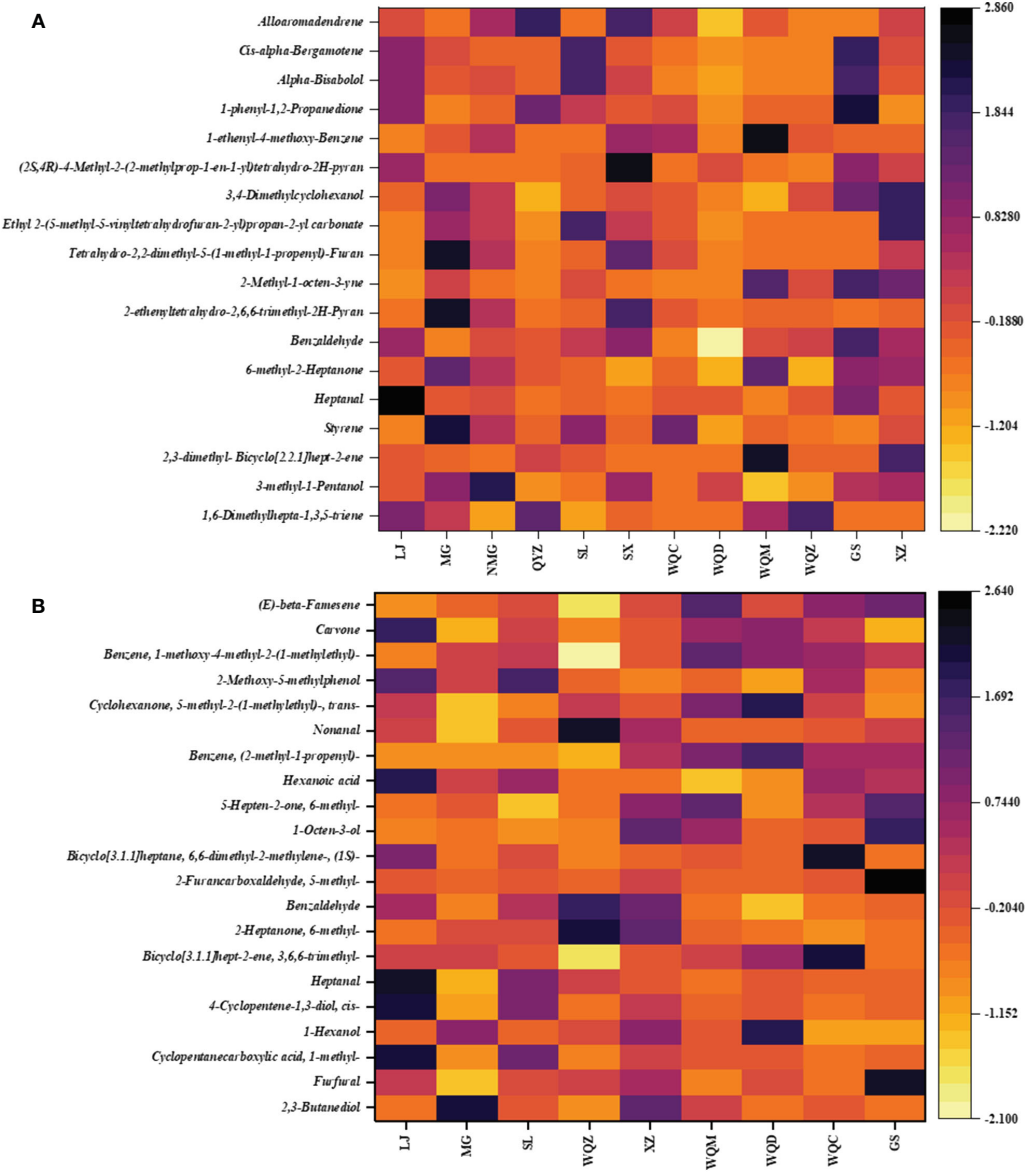
Relative content of differential components in MHS **(A)** and MHR **(B)**.

Higher levels of volatile components, including SX, NMG, WQC, GS, WQZ, QYZ, and WQM, were observed in MHS, while MG, LJ, and XZ exhibited slightly lower levels. These components mainly consist of alkenes and alcohols. Styrene and heptanal groups, especially in XZ, MG, SL, LJ, and Ephedrae herba (as listed in the Chinese Pharmacopoeia), were more abundant among the volatile components. LJ, GS, WQZ, QYZ, and WQM had high levels of benzene, 1-ethenyl-4-methoxy-, 1,2-propanedione, 1-phenyl, *α*-bisabolol, *cis*-*α*-bergamotene, and alloaromadendrene. Bicyclo[2.2.1]hept-2-ene, 2,3-dimethyl- had higher percentages in WQM and XZ, but lower in WQZ, NMG, SX and WQC. The content of styrene group is higher in MG, SX, NMG, WQC, and SL, and lower in WQD, XZ, and QYZ. The heptanal group exhibited the highest percentage in LJ and WQZ. MG had the highest percentage of the 2-heptanone, 6-methyl- group, while other *Ephedra* species had similar percentages. The benzaldehyde group had the lowest content in WQD, with other *Ephedra* species showing similar levels. The 2*H*-pyran, 2-ethenyltetrahydro-2,6,6-trimethyl- group varied most, with MG having the highest content, followed by SX and NMG. The furan, tetrahydro-2,2-dimethyl-5-(1-methyl-1-propenyl)- group was more prominent in MG, SX, and NMG. Ethyl 2-(5-methyl-5-vinyltetrahydrofuran-2-yl) propan-2-yl carbonate had higher relative content in XZ, SL, and MG, but lower in QYZ, LJ, and DZ. The relative content of *cis*-*α*-bergamotene was more pronounced in WQZ, SL, and LJ than in WQD and WQC, while WQM, WQC, and QYZ showed similar levels without significant differences. This analysis is shown in **Figure 4A** and detailed in **Supplementary Table S2**.

WQC, LJ, and GS exhibited higher overall volatile constituent levels among the 8 species of MHR, with notable components including alkenes, aldehydes, and alcohols. In terms of specific differential components, the carvone group showed elevated relative levels in LJ, WQM, WQD, and WQC, while displaying lower levels in MG, LJ, and SL. Conversely, LJ and SL exhibited higher levels of the 2-methoxy-5-methylphenol group compared to other *Ephedra* species. Benzene, 1-methoxy-4-methyl-2-(1-methylethyl)- groups were identified in LJ, MG, SL, WQZ, and XZ, while WQM, WQD, WQC, and GS formed two distinct groups, with the latter showing higher content. Bicyclo[3.1.1]hept-2-ene, 3,6,6-trimethyl-group content varied significantly across all *Ephedra* species, with WQC having the highest relative percentage. Groups such as (*E*)-*β*-famesene, benzenezaldhyde, 1-methoxy-4-methyl-2-(1-methylethyl)-, and cyclohexanone, 5-methyl-2-(1-methylethyl)-, *trans*- exhibited higher levels in the WQC, WQZ, GS, NMG, especially GS. A comprehensive comparison of other groups revealed WQD, WQC, LJ, XZ, and GS as the primary varieties with higher content levels. This analysis is illustrated in **Figure 4B** and detailed in **Supplementary Table S3**.

### 3.2 Analysis of non-volatile components of *Ephedra* using UPLC-Q-TOF-MS

#### 3.2.1 Identification and classification of chemical components via UPLC-MS/MS combined with MN

**Figure 5:**
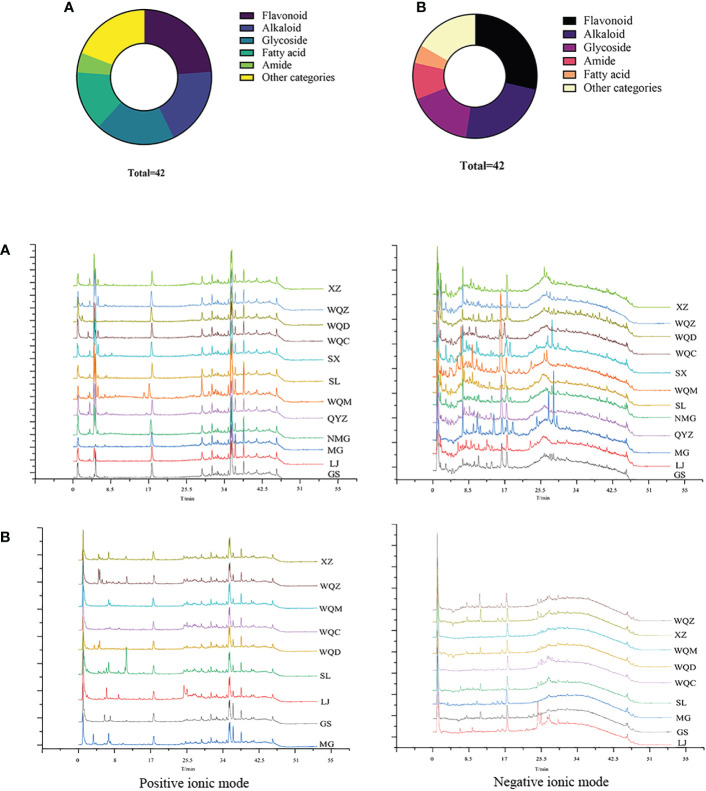
Distribution of chemical component types and total ion flow diagrams in MHS **(A)** and MHR **(B)**.

**Figure 6:**
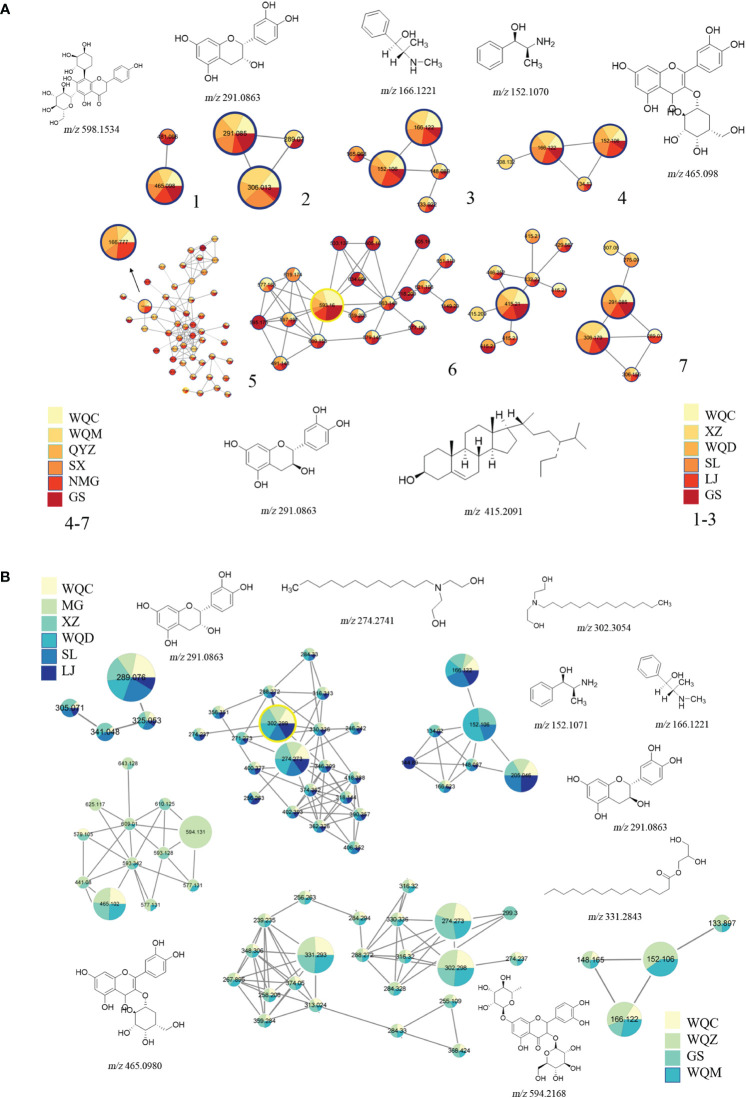
Chemical components identified through molecular networking. **(A)** MHS, **(B)** MHR.

A total of 79 chemical components were identified in 8 species of MHS and MHR, with 42 chemical components present in each species. These components primarily comprised alkaloids, flavonoids, glycosides, carboxylic acids, and fatty acids. MHS contained coumarins, lignans, and monoterpenes, while MHR contained lignans, coumarins, and steroidal compounds. Additionally, alkaloid and flavonoid clusters were predominantly identified in the MN. The distribution of these components is illustrated in **Figure 5** and **Figure 6**, with detailed information provided in **Table 4** and **Table 5**.

**Table 4:** Non-volatile components in MHS.

Peak No.	*t_R_* (min)			Formula	diff(ppm)	Fragment ions (*m/z*)	Identification	Class
1	1.104	183.0863	[M+H]^+^	C_6_H_14_O_6_	-2.32	83.04885;73.0276;71.0472;59.0490;	Dulcitol	Alcohol
2	1.37	130.0495	[M+H]^+^	C_5_H_7_NO	-4.85	130.0495	Ethyl-*p*-methoxycinnamate	Amide
3	1.852	252.0714	[M+H]^+^	C_8_H_13_NO_8_	-1.85	252.0704	Cordycepin	Nucleoside
4	2.052	310.1496	[M+H]^+^	C_12_H_23_NO_8_	-2.05	310.1488;168.1015	Benproperine	Alkaloid
5	2.368	182.1176	[M+H]^+^	C_10_H_12_O_2_[M+NH_4_]^+^	-3.61	182.1168;165.1043;138.0859;134.0958;133.4385;131.0711;125.0507;106.0701;106.9043;	Eugenol	Amide
6	3.316	152.107	[M+H]^+^	C_9_H_13_NO	-1.98	152.1063;117.0694;115.0542;104.0486	Norephedrine	Alkaloid
7	3.665	152.107	[M+H]^+^	C_9_H_13_NO	-2.71	152.1063;134.0958;115.0536;91.0537	*L*-norpseudoephedrine	Alkaloid
8	4.663	166.1226	[M+H]^+^	C_10_H_15_NO	0.08	166.1218;148.1115;133.0873;117.0694;91.0532	Ephedrine	Alkaloid
9	4.929	166.1226	[M+H]^+^	C_10_H_15_NO	-1.48	1661218;148.1114;133.0878;117.0694;104.0609;91.0540	Pseudoephedrine	Alkaloid
10	5.494	180.1383	[M+H]^+^	C_11_H_17_NO	-2.69	180.1375;162.1268;147.1027;141.9559;97.9682;56.9426	Methylephedrine (Tybraine)	Alkaloid
11	7.024	291.0863	[M+H]^+^	C_15_H_14_O_6_	-3.19	552.2261;482.9227;401.2387;193.0851;161.0586	(+)-Catechin	Flavonoid
12	7.374	344.134	[M+H]^+^	C_15_H_18_O_8_[M+NH_4_]^+^	-2.51	291.0851;247.0464;231.0726;207.0645;179.0708;165.0545;147.0439;139.0382;123.0437	Bilobalide	Flavonoid
13	7.806	384.1851	[M+H]^+^	C_13_H_27_N_4_O_9_	1.11	390.1743;314.8123;211.0959;193.0848;161.0590;149.0565;133.0637	(-)-*β*-Hydrastine	Alkaloid
14	8.953	202.0604	[M+H]^+^	C_11_H_6_O_4_[M+NH_4_]^+^	-4.33	384.1849;289.0912;153.0742	Xanthotoxol	Coumarin
15	9.635	291.0863	[M+H]^+^	C_15_H_14_O_6_	5.6	282.1329;184.1087;149.0243;134.0953;117.0695;85.0283	(-)-Epicatechin	Flavonoid
16	12.662	565.1552	[M+H]^+^	C_26_H_28_O_14_	-1.78	207.0649;189.0523;179.0692;165.0534;147.0431;139.0385;123.0433;111.0427;	Vicenin-1	Flavonoid
17	13.81	565.1552	[M+H]^+^	C_26_H_28_O_14_	-4.22	565.1515;547.1434;529.1305;511.1180;481.1168465.6435;445.1041;427.0985;409.0878;391.0783;355.0787;337.0698;307.0391	Schaftoside	Flavonoid
18	17.335	464.0955	[M+H]^+^	C_21_H_20_O_12_	-0.86	303.0484	Hyperoside (IS)	Glycoside
19	23.008	358.1847	[M+H]^+^	C_17_H_24_O_7_	1.71	179.2667	Nebrodenside A	Glycoside
20	30.54	274.2741	[M+H]^+^	C_16_H_35_NO_2_	-2.47	274.2729;256.2618;230.2492;106.0853;88.0751;57.0699	Hexadecanoic acid	Fatty acid
21	31.771	302.304	[M+H]^+^	C_16_H_37_N_4_O	2.23	302.3042;284.2879;240.2616;106.0861;88.0750;70.0648	Stearic acid	Fatty acid
22	31.987	415.2091	[M+H]^+^	C_22_H_32_O_6_[H+Na]^+^	4.45	415.2089;135.0800;119.0850;107.0855;91.0537	*β*-Sitosterol	Alcohol
23	33.251	330.3367	[M+H]^+^	C_20_H_43_NO_2_	-1.36	330.3352;312.3209;286.3096;265.0108;238.9868;168.0762;149.0592;124.0845;106.0849;88.0765	Erucylamide	Fatty acid
24	1.105	379.1035	[M-H]^-^	C_17_H_18_O_7_[M+HCOO]^-^	-2.26	379.1298;181.0746;161.0480;143.0375;119.0365;101.0257;89.0254	Byakangelicin	Coumarin
25	1.355	191.0224	[M-H]^-^	C_9_H_6_NO_4_	-2.58	290.0924;230.0752;200.0596	Damascenone	monoterpene
26	2.037	327.0887	[M-H]^-^	C_16_H_12_O_4_[M+CH_3_COO]^-^	-1.21	327.0904;291.1126	Formononetin	Flavonoid
27	2.619	593.1373	[M-H]^-^	C_23_H_20_N_11_O_9_	-0.64	593.1402;575.1266	Vicenin-2	Glycoside
28	2.868	315.0748	[M-H]^-^	C_16_H_14_NO_6_	2.83	315.0771;164.8337;152.0122;108.0227	5,7-Dihydroxy-3’,4’-dimethoxyflavanone	Flavonoid
29	3.068	305.0694	[M-H]^-^	C_18_H_12_NO_4_	1.12	305.0715;177.0576;167.0368;13.0419;135.0418;123.0100;121.0296;111.0439;95.0508;83.0148;	(-)-Epigallocatechin	Flavonoid
30	3.467	315.0748	[M-H]^-^	C16H14NO6	1.52	315.0771;153.0218;109.0306	Linalool 3-*O*-*β*-D-glucopyranoside	Glycoside
31	6.011	895.1822	[M-H]^-^	C_34_H_38_C_l2_N_10_O_15_	0	895.1865;812.1753;727.1405;467.1037;427.0748;289.0760	22-Acetoxyl licorice saponin	Glycoside
32	7.342	325.0956	[M-H]^-^	C_18_H_16_NO_15_	-1.09	325.0925	*O*-Coumaric acid glucoside	Glycoside
33	7.924	365.1507	[M-H]^-^	C_21_H_22_N_2_O_4_	-0.66	365.1509;221.1054	Dehydrocorydaline	Alkaloid
34	8.689	327.1112	[M-H]^-^	C_18_H_18_NO_5_	3.35	327.1133;162.8381;147.0466;101.0256;	(10E,15E)-9,12,13-trihydroxyoctadeca-10,15-dienoic acid	Fatty acid
35	9.454	289.0747	[M-H]^-^	C_15_H_14_O_6_	2.65	289.0762;245.0850;221.0867;203.0733;179.0362;151.0427;125.0265;125.0257;109.0305;	Cianidanol	Flavonoid
36	9.72	287.07	[M-H]^-^	C_17_H_10_N_3_O_2_	1.14	287.0720;271.0669;245.0849	2’-Hydroxy-a-naphthoflavone	Flavonoid
37	10.635	593.1584	[M-H]^-^	C_20_H_24_N_11_O_11_	-0.62	593.1598;503.1269;473.1183;455.1050;437.0912;383.0828;353.0717;325.0736	Apigenin-6,8-C-dihexoside	Glycoside
38	16.023	295.0486	[M-H]^-^	C_16_H_10_NO_5_	2.23	295.0503;173.0110;155.0007;129.0212;111.0103;101.0253	Phytol	Fatty acid
39	17.326	465.1028	[M-H]^-^	C_21_H_20_O_12_	-0.76	465.1017;305.0548;304.0526;303.0490;91.0387	Hyperoside (IS)	Glycoside
40	17.969	577.1635	[M-H]^-^	C_20_H_24_N_11_O_10_	-0.41	577.1661;457.1195;413.0937;341.0705;323.0598;293.0499	Vitexin-2-*O*-rhamnoside	Glycoside
41	22.659	463.0954	[M-H]^-^	C_14_H_14_N_11_O_8_	-2.17	463.0948;301.0387;302.0405	Quercetin-4’-*O*-glucoside	Glycoside
42	26.85	453.2301	[M-H]^-^	C_28_H_22_O_6_	1.35	399.8592;358.0988;330.0988;328.1359;295.0684;253.0510;77.0404	Epsilon-viniferin	Others
43	27.648	357.1946	[M-H]^-^	C_20_H_22_O_6_	4.54	161.049	Matairesinol	Fatty acid
44	43.132	393.2804	[M-H]^-^	C_28_H_58_	3.89	393.2841;376.0649;358.7872;342.7539;293.3779;239.0405;214.8533;197.6492;116.9290	Octacosane	Fatty acid

**Table 5:** Non-volatile components in MHR.

Peak No.	*t_R_* (min)			Formula	diff(ppm)	Fragment ions (*m/z*)	Identification	Class
1	1.637	294.1547	[M+H]^+^	C_12_H_20_O_7_, [M+NH4]^+^	-1.08	294.1544;213.1344;212.1271;170.1149	Anastrozole	Others
2	2.036	310.1496	[M+H]^+^	C_12_H_23_NO_8_	-1.84	310.1476;293.1152;147.0433;	Macusine B	Alkaloid
3	2.423	328.1391	[M+H]^+^	C_15_H_18_O_7_, [M+NH4]^+^	-0.67	328.1379;311.1312;132.0798	4-*β*-D-glucopyranosyloxy-trans-Cinnamaldehyde	Glycoside
4	3.479	427.2061	[M+H]^+^	C_18_H_28_N_5_O_7_	1.24	427.2067;177.0537;145.0280;117.0323;89.0386	2’-Deoxyguanosine-5’-diphosphate	Glycoside
5	3.663	224.1281	[M+H]^+^	C_12_H_14_O_3_, [M+NH_4_]^+^	-2.53	224.1281;121.0633;88.1977	Ethyl-*p*-methoxycinnamate	Amide
6	4.059	450.1871	[M+H]^+^	C_21_H_27_N_3_O_8_	-1.22	450.1857;177.0540;145.0277;112.0865	Cyanidin-3-*O*-galactoside	Glycoside
7	4.639	166.1221	[M+H]^+^	C_10_H_15_NO	-3.06	166.1221;103.0535;91.0536;78.0452;77.0372	Ephedrine	Alkaloid
8	4.955	166.1226	[M+H]^+^	C_10_H_15_NO	-2.88	166.1222;148.1077;132.0803;115.0539;91.0538	Pseudoephedrine	Alkaloid
9	5.483	180.1383	[M+H]^+^	C_11_H_17_NO	-2.04	180.1379;162.1277;147.1033;117.0700	Methylpseudoephedrine	Alkaloid
10	5.879	523.2902	[M+H]^+^	C_27_H_36_N_7_O_4_	-1.5	523.2902	Ephedradine B/D	Alkaloid
11	6.037	493.2809	[M+H]^+^	C_28_H_36_N_4_O_4_	-0.82	493.2755;476.2544;419.1944;348.1464;323.1430;265.0846;237.0907;212.1739;198.1591;	Ephedradine A	Alkaloid
12	6.986	553.3007	[M+H]^+^	C_21_H_20_N_2_O_3_	-1.05	177.0542;145.0278;117.0334	*N*-trans-feruloylputrescine	Alkaloid
13	7.356	288.1343	[M+H]^+^	C_15_H_17_N_3_O_3_	-1.76	288.1333;177.0540	Feruloylhistamine	Alkaloid
14	7.883	609.1225	[M+H]^+^	C_28_H_22_N_3_O_13_	-0.26	609.1222;441.0808;303.0491	Gallocatechin-(4 → 6″; 2 → *O* → 7″)-epigallocatechin	Flavonoid
15	8.279	594.2168	[M+H]^+^	C_26_H_33_N_4_O_12_	0.41	594.2164;317.1017;151.0384	Kaempferol-3-*O*-glucoside-7-*O*-rhamnoside	Glycoside
16	8.912	282.1336	[M+H]^+^	C_14_H_16_O_5_, [M+NH_4_]^+^	-1.59	282.1329;134.0952;85.0279;57.0338	1-Methladenosine	Others
17	9.255	467.1548	[M+H]^+^	C_22_H_26_O_11_	-2.04	467.1538;305.0999	Curculigoside	Glycoside
18	9.492	291.0863	[M+H]^+^	C_15_H_14_O_6_	-0.65	291.0858;165.0599;139.0385	(+)-Catechin	Flavonoid
19	10.785	595.1646	[M+H]^+^	C_18_H_36_N_5_O_11_S_3_	0.01	595.1646	Vicenin-2	Amide
20	11.101	567.3164	[M+H]^+^	C_30_H_46_O_10_	0.69	567.3124;550.2906;493.2318;422.1642;339.1218;325.1061;229.2014;198.1595;	4-Ketozeinoxanthin	Terpene
21	12.552	275.0914	[M+H]^+^	C_15_H_14_O_5_	-2.33	169.0473;151.0359;127.0414;121.0646;107.0487	Phloretin	Flavonoid
22	14.055	305.102	[M+H]^+^	C_16_H_16_O_6_	-1.44	305.1013;221.0805;193.0847;147.0427;139.0379;137.0593	Oxypeucedaninhydrate	Coumarin
23	15.136	291.0975	[M+H]^+^	C_14_H_14_N_2_O_5_	-0.49	291.0968;273.0900;227.0808;188.0696;170.0593;159.0902;130.0646;115.0516;87.0068	Epigallocatechin	Flavonoid
24	17.326	465.1028	[M+H]^+^	C_21_H_20_O_12_	-0.76	465.1017;305.0548;304.0526;303.0490;91.0387	Hyperoside (IS)	Flavonoid
25	23.629	343.1176	[M+H]^+^	C_19_H_18_O_6_	-1.03	343.1176;344.1204;345.1213	Methylophiopogonanone A	Flavonoid
26	24.315	545.1429	[M+H]^+^	C_28_H_22_N_3_O_9_	0.24	545.1429	Mahuangnin A/B/C1	Flavonoid
27	24.684	545.1429	[M+H]^+^	C_27_H_16_N_10_O_4_	0.73	545.1429	Mahuangnin A/B/C2	Flavonoid
28	25.027	545.1429	[M+H]^+^	C_28_H_22_N_3_O_9_	1.19	545.1429	Mahuangnin A/B/C3	Flavonoid
29	26.689	543.1286	[M+H]^+^	C_30_H_22_O_10_	-0.96	543.1275;525.1154;497.1149;419.0764;407.0759;379.0788	Mahuannin F	Flavonoid
30	27.083	357.1333	[M+H]^+^	C_20_H_20_O_6_	-0.97	357.1325;327.1207;165.0538;151.0377;137.0593;	Sauchinone	Others
31	27.982	529.148	[M+H]^+^	C_28_H_22_N_3_O_8_	1.14	529.1463;403.1181;393.0965;267.0637;255.0637	Mahuannin D	Flavonoid
32	28.852	348.2744	[M+H]^+^	C_17_H_31_N_8_	-1.38	348.2738;314.035;277.2172;195.1369;155.1059;135.1146;109.0997;	Vanillicacid1-*O*-glucopyranoside	Glycoside
33	29.934	225.1961	[M+H]^+^	C_13_H_24_N_2_O	-1.46	225.1950;193.1581;165.1593;149.1266;	Sinapinic acid	Amide
34	30.54	274.2741	[M+H]^+^	C_16_H_35_NO_2_	-0.87	274.2729;256.2623;230.2470;106.0854;88.0754;	Lauryldiethanolamine	Alkaloid
35	31.776	302.3054	[M+H]^+^	C_18_H_39_NO_2_	-1.49	302.3048;284.2932;106.0857;88.0755	Tetradecyldiethanolamine	Alkaloid
36	31.964	415.2091	[M+H]^+^	C_22_H_32_O_6_	4.84	415.2091;133.0638;119.0851;107.0850;91.0540	Austinoneol	Terpene
37	33.257	330.3367	[M+H]^+^	C_20_H_43_NO_2_	-1.18	330.3292;312.3244;136.8741;119.0825;106.0858;88.0756;70.0651	Europine	Alkaloid
38	39.323	256.2635	[M+H]^+^	C_16_H_33_NO	-1.6	256.2627;165.0854;	Diphenydramine	Alkaloid
39	40.37	331.2843	[M+H]^+^	C_19_H_38_O_4_	-0.61	331.2835;313.2724;257.2460;239.2352;151.2472;137.1321;123.1161;109.1005;95.0851;	1-Hexadecanoyl-sn-glycerol	Amide
40	9.232	501.1255	[M-H]^-^	C_28_H_35_FO_7_	0.29	501.1257;465.1459;303.0925	Amcinonide	Others
41	10.682	281.072	[M-H]^-^	C_19_H_10_N_2_O	-2.04	281.0690;193.0538;178.0276;149.0623;134.0387	Feruloyllactate	Amide
42	17.173	463.0882	[M-H]^-^	C_21_H_20_O_12_	0.94	461.0803;463.0882;464.0988;465.1011	Hyperoside (IS)	Glycoside
43	28.014	563.12	[M-H]^-^	C_26_H_28_O_14_	0.25	563.1197;527.1449;401.1089;391.0919;273.0797	Isoschaftoside	Glycoside
44	40.321	279.2388	[M-H]^-^	C_10_H_30_N_7_O_2_	-3.69	280.2142;279.2368	10*E*,12*Z*-Linoleic acid	Fatty acid

##### 3.2.1.1 Identification of alkaloids

Alkaloids in MHS encompass methamphetamine alkaloids, benzoylisoquinoline alkaloids and benzylisoquinoline alkaloids. Methamphetamine alkaloids (17,18,19) include norephedrine (*m/z* 152.1070, [M+H]^+^), *l*-norpseudoephedrine (*m/z* 152.1070, [M+H]^+^), ephedrine (*m/z* 166.1223, [M+H]^+^), methylephedrine (*m/z* 180.1383, [M+H]^+^), and pseudoephedrine (m/z 166.1223, [M+H]^+^). These typically undergo cleavage individually through the loss of groups like -H_2_O, -NH_2_, and -CH_3_, or through paired cleavage forming a stable conjugated double-bonded state (**Supplementary Figure S2**). Isoquinoline alkaloids include benproperine (*m/z* 310.1496, [M+H]^+^), inferred from secondary fragment ions at *m/z* 310.1488 and 168.1015, and (-)-*β*-hydrastine (*m/z* 384.185, [M+H]^+^), deduced from secondary fragment ions at *m/z* 384.1849, 289.0912, and 153.0742.

Alkaloids in MHR consist of macrocyclic spermine alkaloids, tyramine alkaloids, and alcoholamine alkaloids. Macrocyclic arginine alkaloids (Lv et al., 2015) include ephedradine B/D (*m/z* 523.2902, [M+H]^+^) and ephedradine A (Guo, 2021) (*m/z* 493.2809, [M+H]^+^), typically undergoing cleavage either individually or in pairs through groups such as -H_2_O, -NH_2_, -CH_3_. Compared to alkaloids in MHS, MHR alkaloids can eliminate the side-chain moiety during cleavage, as shown in **Supplementary Figure S3**. Tyramine alkaloids include c*is*-*N*-feruloylputrescine (*m/z* 553.3007, [M+H]^+^), inferred from secondary fragment ions at *m/z* 177.0542, 117.0334, and *m/z* 145.0278, 117.0334, and feruloylhistamine (*m/z* 288.1343, [M+H]^+^) (Lv et al., 2015). Alcoholamine alkaloids include lauryldiethanolamine (*m/z* 274.2741, [M+H]^+^), tetradecyldiethanolamine (*m/z* 302.3054, [M+H]^+^), and diphenhydramine (*m/z* 256.2635, [M+H]^+^), identified through MN and comparison of their secondary fragment ions.

##### 3.2.1.2 Identification of flavonoids

Ten flavonoids were tentatively identified in MHS, including (+)-catechin (*m/z* 291.0863, [M+H]^+^) (Lv et al., 2015) and (-)-epicatechin (*m/z* 289.0747 [M-H]^-^) (Khattabi et al., 2022). Cleavage of these compounds typically involves the removal of groups such as -H_2_O and -CO_2_. Additionally, bilobalide (*m/z* 291.0863, [M+H]^+^), formononetin (*m/z* 327.0887, [M-H]^-^), and byakangelicin (*m/z* 379.1035, [M-H]^-^) were identified using the Agilent herbal library-v20–04-17. Other components include schaftoside (*m/z* 565.1552, [M+H]^+^) (Li et al., 2022) and 5,7-dihydroxy-3’,4’-dimethoxyflavanone (*m/z* 315.0748, [M-H]^-^), deduced from secondary fragment ions at *m/z* 315.0771, 164.8337, 152.012, and 108.0227. (-)-Epigallocatechin (*m/z* 305.0694, [M-H]^-^) (Khattabi et al., 2022) and cyanidanol (*m/z* 289.0747, [M-H]^-^) were identified, with secondary fragment ions observed at *m/z* 289.076, 245.0850, 221.0867, 203.0733, 179.0362, 151.0427, 125.0265, 125.0257, and 109.0305. Finally, 2’-hydroxy-*α*-naphthoflavone (*m/z* 287.0700, [M-H]^-^) was deduced from secondary fragment ions at *m/z* 287.0720, 271.0669, and 245.0849.

Flavonoids were also tentatively identified in MHR, including gallocatechin- (4 → 6″; 2 → O → 7″)-(epi)gallocatechin (*m/z* 609.1225, [M+H]^+^), (+)-catechin (*m/z* 291.0863, [M+H]^+^), phloretin (*m/z* 275.0914, [M+H]^+^), catechina/(epi)gallocatechin (*m/z* 291.0975, [M+H]^+^), methylophiopogonanone A (*m/z* 343.1176, [M+H]^+^), mahuangnin A/B/C1, 2, 3 (*m/z* 545.1429, [M+H]^+^), mahuannin F (*m/z* 543.1286 [M+H]^+^), and mahuannin D (*m/z* 529.148 [M+H]^+^) (Al-Rimawi et al., 2017; Li et al., 2017; Guo, 2021; Li et al., 2022).

##### 3.2.1.3 Identification of fatty acids

Fatty acids tentatively identified in MHS include hexadecanoic acid (*m/z* 274.2741, [M+H]^+^) and (10*E*,15*E*)-9,12,13-trihydroxyoctadeca-10,15-dienoic acid (*m/z* 327.1112, [M-H]^-^), determined through MN analysis and comparison of secondary fragment ions. Additionally, stearic acid (*m/z* 302.304, [M+H]^+^) was identified based on secondary fragment ions at *m/z* 302.3042, 284.2879, 240.2616, 106.0861, 88.0750, and 70.0648. Erucylamide (*m/z* 330.3367, [M+H]^+^), phytol (*m/z* 295.0486, [M-H]^-^), and octacosane (*m/z* 393.2804, [M-H]^-^) were also identified through comparison using TCMSP.

In MHR, two fatty acids were tentatively identified: 1-hexadecanoyl-sn-glycerol (*m/z* 331.2843, [M+H]^+^) and 10*E*, 12*Z*-linoleic acid (*m/z* 279.2388, [M-H]*-*), determined through MN and comparison of their secondary fragment ions.

##### 3.2.1.4 Identification of flavonoids glycosides

Flavonoids glycosides identified in MHS include nebrodenside A (*m/z* 358.1847, [M+H]^+^) (Cottiglia et al., 2005), vicenin-2 (*m/z* 593.1373, [M-H]^-^) (Qi et al., 2014; Guo, 2021; Li et al., 2022), linalool 3-*O*-*β*-D-glucopyranoside (*m/z* 315.0748, [M-H]^-^), with secondary fragment ions observed at *m/z* 315.0771, 153.0218, and 109.0306. Aditionally, apigenin-6,8-*C*-dihexoside (*m/z* 593.1584, [M-H]^-^) (Xia et al., 2017; Li et al., 2022), vitexin-2 (*m/z* 593.1584, [M-H]^-^) (Guo, 2021; Li et al., 2022), vitexin-2-*O*-rhamnoside (*m/z* 577.1635, [M+H]^+^) (Guo, 2021; Li et al., 2022), and quercetin-4’-*O*-glucoside (*m/z* 463.0954, [M-H]^-^) (Li et al., 2022).

The flavonoid glycosides identified in MHR include cyanidin-3-*O*-galactoside (*m/z* 450.1871, [M+H]^+^) and kaempferol-3-*O*-glucoside-7-*O*-rhamnoside (*m/z* 609.1225, [M+H]^+^), determined through MN and comparison of their secondary fragment ions. Curculigoside (*m/z* 467.1548, [M+H]^+^) was inferred from the secondary fragment ions observed at m/z 467.1538 and 305.0999.

#### 3.2.2 Chemometric analyses

**Figure 7:**
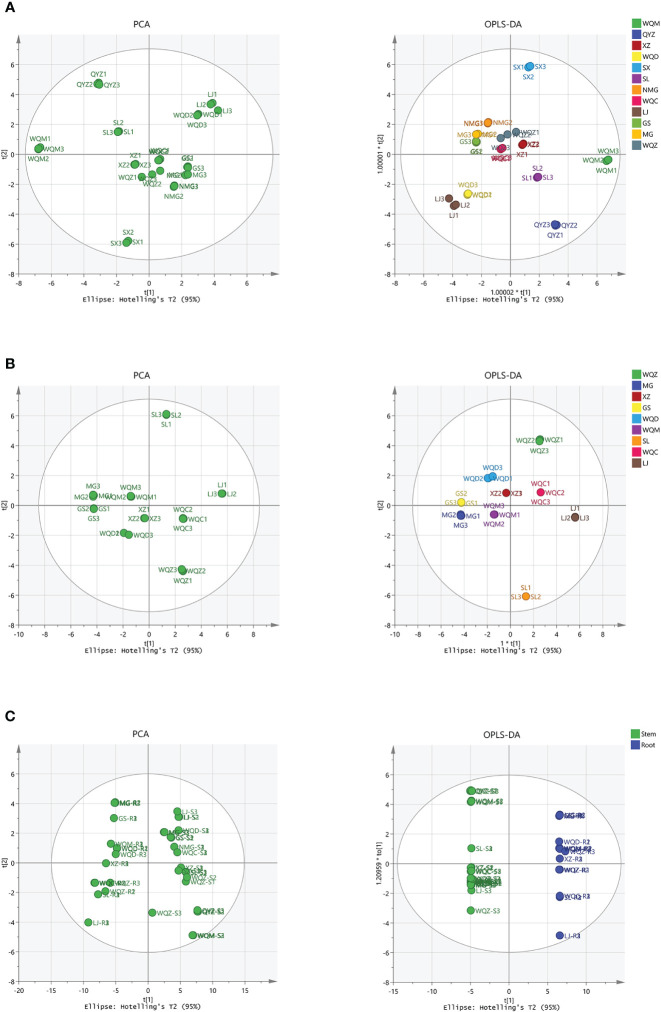
Multivariate statistical analysis, including PCA and OPLS-DA, was conducted on the UPLC-MS data of non-volatile components. **(A, B)** represent the analysis of MHS and MHR, respectively, with R^2^X and R^2^Y values of 0.937, 0.727 for MHS, and 0.887 and 0.761 for MHR. **(C)** displays PCA and OPLS-DA results (R^2^X, R^2^Y values of 0.916, 0.991) for MHS and MHR.

PCA was initially conducted on the samples, revealing a distinct separation between the varieties. Subsequently, supervised OPLS-DA with a confidence interval of 95% was performed based on the PCA results. This analysis indicated a significant separation among the varieties, supported by parameter indices R2X > 0.5 and R2Y > 0.5, demonstrating the robustness and reliability of the OPLS-DA model. Moreover, the distribution of non-volatile components among different varieties of *Ephedra* plants exhibited considerable variability, as they were dispersed in distinct regions. Furthermore, a comparison of non-volatile components between stems and roots revealed substantial differences between these two parts. In total, 18 and 21 differential components were identified in 8 species of MHS and MHR, respectively, with variable importance in projection (VIP) scores > 1 and significance level (P) < 0.05, as illustrated in **Figure 7** and **Supplementary Table S4**.

#### 3.2.3 Comparative analysis of the relative abundance of major chemical constituents and differentiated components

**Figure 8:**
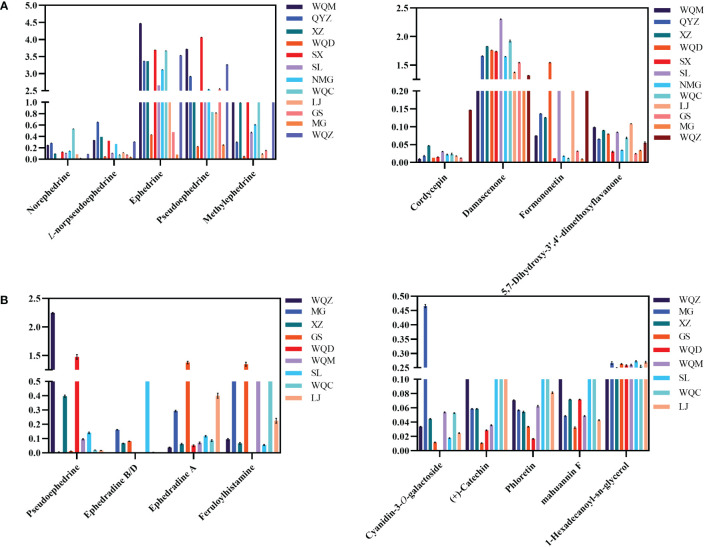
Relative abundance of major chemical constituents and differentiated components in MHS **(A)** and MHR **(B)**.

UPLC-MS analysis detected five phenylpropane alkaloids—norephedrine, l-norpseudoephedrine, ephedrine, pseudoephedrine, and methylephedrine—in the extracts of 8 species of MHS. Relative abundance analysis revealed elevated levels of these alkaloids in SX, NMG, GS, WQ, and QYZ, whereas SL, LJ, XZ, WQD, and MG exhibited lower contents. Additionally, cordycepin, damascenone, formononetin, and 5,7-dihydroxy-3’,4’-dimethoxyflavanone were identified as differential components in the non-alkaloidal fraction. Comparative analysis of the total content across the four species indicated that SL, WQD, and LJ were predominant, with the remaining *Ephedra* species showing relatively lower levels, as depicted in **Figure 8A**.

Macrocyclic spermine alkaloids, including ephedradine A, B, D, and feruloylhistamine, were identified in MHR. The alkaloidal fraction of the eight MHR species contained differential components such as pseudoephedrine, ephedrine B/D, and ephedrine A. Regarding total relative abundance, WQZ, GS, WQD, and WQC exhibited the highest levels, followed by MG, XZ, and SL. Non-alkaloidal differential components included cyanidin-3-*O*-galactoside, (+)-catechin, phloretin, and mahuannin F. Comparisons revealed dominance by WQZ, GS, WQC, MG, and SL, while the relative abundances in other *Ephedra* species ranged more evenly between 0.3 and 0.4. Interestingly, cyanidin-3-*O*-galactoside was most abundant in MG, along with WQZ, GS and XZ, which not only had higher total amounts but also higher content of each component within them, as shown in **Figure 8B**.

### 3.3 MetaboAnalyst and kyoto encyclopedia of genes and genomes enrichment analysis

#### 3.3.1 Biogenic synthesis pathway of volatile components

**Figure 9:**
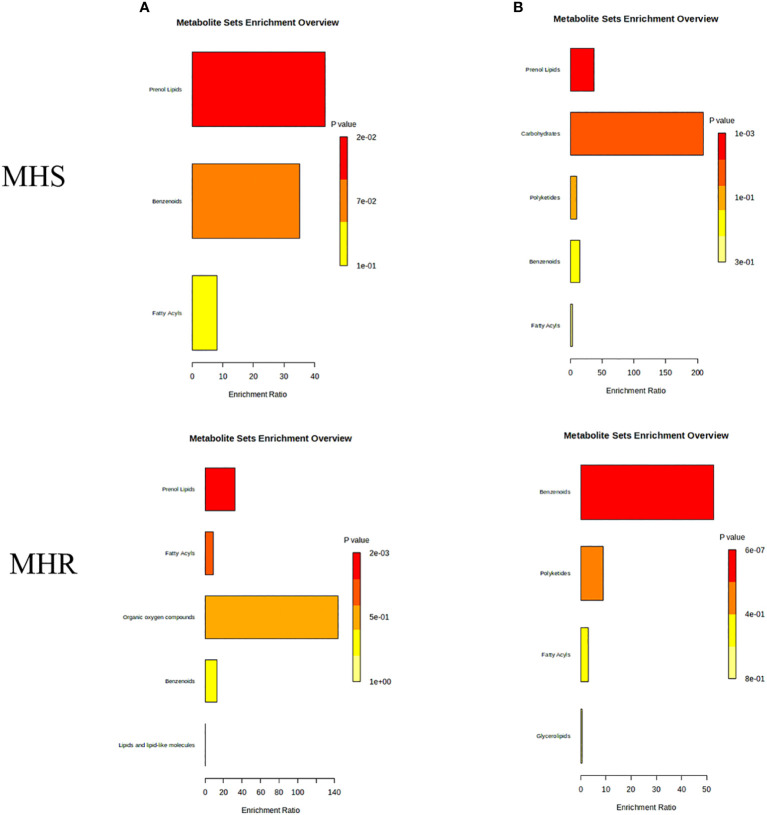
Synthetic pathways enriched with differential components.

The analysis of the differential components was conducted using MetaboAnalyst, revealing enriched synthetic pathways such as fatty acyl synthesis, phenylpropane synthesis (including lignan and flavonoid pathways), terpene and steroid biosynthesis, lipids and lipoidal synthesis, and organic oxygen component synthesis pathways, as illustrated in **Figure 9A**. These pathways predominantly involve enrichment to olefins, acids, alcohols, and terpenoids. The fatty acids pathways mainly involve map 01120 (microbial metabolism in diverse environments) and map 01062 (biosynthesis of terpenoids and steroids). In addition, the KEGG pathway was enriched with heptanal, caproic acid, (*R*)-1-octen-3-ol, *α*-bisabolol, (*E*)-*β*-famesene, and nonanal, with higher content observed in the stems and roots of LJ and GS, compared to SL roots. Additionally, in the monoterpene biosynthetic pathway, KEGG annotation to Carvone was identified, belonging to isoprenoid (monoterpene alkene) synthesis.

#### 3.3.2 Biogenic synthesis pathway of non-volatile components

The analysis of non-volatile differential components identified enriched synthetic pathways, such as lipid synthesis, isoprene (diterpene) synthesis, benzene synthesis, and sugar metabolism pathways, as depicted in **Figure 9B**. These pathways were primarily enriched with alkaloids, flavonoids, and terpenoids. Alkaloids were notably involved in map 00996 (biosynthesis of various alkaloids), map 01063 (biosynthesis of alkaloids derived from the shikimate pathway), map 01100 (metabolic pathways), and map 01110 (biosynthesis of secondary metabolites), with KEGG annotations to norephedrine and pseudoephedrine. These alkaloids were found higher levels in WQC, WQZ, WQM, and SX. Flavonoids were predominantly involved in map 00941 (flavonoid biosynthesis), map 01061 (biosynthesis of phenylpropanoids), map 01100 (metabolic pathways), and map 01110 (biosynthesis of secondary metabolites), with KEGG annotations to 5,7-dihydroxy-3’,4’-dimethoxyflavanone, epicatechin, and cyanidin-3-galactoside. These flavonoids were found in higher amounts in WQC, WQM, SL, and MG.

## 4 Discussion

*Ephedra* is widely used in clinical applications, with *E. sinica* Stapf, *E. intermedia* Schrenk et C. A. Mey, and *E. equisetina* Bge. recognized as official varieties in the Chinese Pharmacopoeia. Despite originating from the same botanical source, MHS and MHR exhibit distinct pharmacological effects, known as “Same source, Different effect.” This variation is attributed to differences in their chemical components, notably the presence of methamphetamine alkaloids predominantly in MHS and macrocyclic arginine alkaloids in MHR. China boasts abundant medicinal resources of *Ephedra* plants, making it a significant commodity in the import and export of Chinese herbal medicine.

In this study, significant differences in chemical components among different parts of Ephedra plants were observed. Volatile components in *Ephedra* stems primarily include aldehydes, olefins, and alcohols. In contrast, *Ephedra* roots contain olefins, terpenes, and aldehydes. The relative content of volatile components is higher in *Ephedra* stems than in roots. Non-volatile components in Ephedra stems mainly consist of isoquinoline alkaloids, phenylpropanoid alkaloids, flavonoids, flavonoid glycosides, and organic acids, whereas *Ephedra* roots predominantly contain macrocyclic arginine alkaloids, tyramine alkaloids, alcohol amine alkaloids, bis-flavonoids, and a small amount of amino acids and lignans. These differences in chemical constituents contribute to distinct medicinal effects.

*Ephedra* alkaloids exert sympathomimetic effects, dilate bronchioles, induce vasoconstriction, and stimulate the central nervous system (Chen et al., 2010). *Ephedra* stems are traditionally used to dispel wind-cold, elevate blood pressure, promote lung function, and relieve asthma symptoms, treating conditions such as influenza, cough, and chest oppression. On the other hand, *Ephedra* root are employed to regulate sweating, secure the exterior, and lower blood pressure, commonly prescribed for conditions characterized by spontaneous perspiration and night sweats (Hui; Li et al., 2018). The diverse therapeutic effects of *Ephedra* stem and root arise from the presence of alkaloids, flavonoids, and volatile oils. Ephedrine in *Ephedra* stems induces sweating (Liao et al., 2015), mahuannin B in roots have the opposite effect (Wang et al., 2017).

In addition to the legally recognized medicinal MHS, SL, XZ, MG, and LJ exhibited relatively high content compared to other varieties in this study. Although their sweating effect may be slightly weaker than that of legally recognized *Ephedra* stems, their use can be considered with dosage adjustment. Macrocyclic arginine alkaloids in *Ephedra* root, such as ephedradine B and epicatechin, exhibit antihypertensive effects, significantly reducing both systolic and diastolic blood pressure (Yanfang et al., 2010). Besides the legally recognized *Ephedra* root, species such as XZ, MG, WQM, and LJ could also serve as medicinal alternatives based on the comparison of their primary chemical components. Notably, the relative content of ephedrine in species WQM is higher than that of the legally recognized variety. The “Same Source, Different Effects” phenomenon in *Ephedra* primarily arises from variations in the chemical composition of alkaloids and flavonoids.

Phylogenetically closely related species often share similar chemical profiles and clinical efficacy, making them valuable medicinal herbs. This study compared the volatile and non-volatile components of eight *Ephedra* species. It identified olefins, alcohols, aldehydes, and ketones as volatile compounds, and alkaloids, flavonoids, carboxylic acids, and fatty acids as non-volatile compounds. Variations in chemical composition were attributed to differences in species and origins. The legally recognized *Ephedra* exhibited the highest content, followed by MG, XZ, and LJ. These differences in content ultimately lead to variations in their biosynthetic pathways.

Differential volatile components are enriched in pathways like fatty acyl synthesis, phenylpropane synthesis, terpenoid and steroid biosynthesis, lipid and lipid-like component synthesis, and organic oxygen species synthesis. Fatty acyl synthesis, a crucial pathway in fatty acid production, serves as a primary energy source for organisms. The structure and properties of biological membranes are linked to the cold tolerance of plants. Low temperatures enhance fatty acid desaturase enzyme activity, increasing levels of unsaturated fatty acids. This reduces membrane lipid saturation and enhances membrane fluidity, stabilizing plant growth in cold conditions and ultimately improving cold tolerance (Sun et al., 2023). The fatty acid synthesis pathway is enriched with differential metabolites of MHS (heptanal) and MHR (heptanal, caproic acid, (*R*)-1-octen-3-ol). Heptanal and (*R*)-1-octen-3-ol are more abundant in LJ and GS stems and roots, mainly distributed in Yunnan, Gansu, and Tibet of China, regions characterized by lower average annual temperatures and higher altitudes. These low-temperature conditions may enhance the synthesis of heptanal and (*R*)-1-octen-3-ol, promoting stable growth in cold environments. Benzaldehyde, found in *Ephedra* stem and root, is part of the phenylpropane metabolic synthesis pathway. Benzaldehyde synthesis involves three metabolic pathways: toluene degradation, aminotoluic acid degradation, and aromatic component degradation. The lignan and flavonoid pathways play crucial roles in phenylpropane metabolism. Lignan primarily accumulating in plant secondary cell walls, providing mechanical support, conduits for water and mineral transport, participating in other development processes, resisting pathogenic, and enhancing resistance to abiotic stresses. The isopentenol ester synthesis pathway is also involved in fatty acid synthesis (Sun et al., 2023).

The enriched synthetic pathways for non-volatile differential components encompass lipid synthesis, isoprene (diterpene) synthesis, benzene synthesis, and sugar metabolism pathways. The diverse structures of lipids contribute to various crucial biological functions. Lipids are the primary constituents of biological membranes and participate in signaling, regulation of cell growth, differentiation, senescence, programmed cell death, and other cellular processes. Additionally, they provide energy for growth and support vital activities. Polyketide synthesis is part of lipid synthesis (Sun et al., 2023). For example, formononetin and 5,7-dihydroxy-3’,4’-dimethoxyflavanone from MHS, and epicatechin and phloretin from the root, participate in the synthesis of isoflavones, flavonoids, and phenylpropanoids. Formononetin, known for its antioxidant, antihypertensive, antitumour, and anti-infection properties (Yang et al., 2021), and epicatechin, recognized for its antioxidant activity and hypolipidemic effects, as well as its potential to mitigate oxidative stress damage (Yanagimoto et al., 2023), are flavonoid compounds. Studies have demonstrated their efficacy in antitumor, anti-inflammatory, hypolipidemic, and antihypertensive effects. The benzene synthesis pathway is enriched with norephedrine and pseudoephedrine in *Ephedra* stems, and ephedrine and cyanidin-3-galactoside in roots. This pathway contributes to the biosynthesis of alkaloids, such as mangiferic acid derivatives. Therefore, the pathways for the biogenic synthesis of differential components in *Ephedra* stems and roots may vary depending on the variety, origin, and harvest time. This variability in pathways may be influenced by factors such as origin, variety, and harvest time (Cui et al., 2020), as shown in **Supplementary Figure S5**. Consequently, differential synthesis is observed. According to the Chinese Pharmacopoeia (2020 edition), the roots of *E. sinica* Stapf and *E. intermedia* Schrenk et C. A. Mey are designated as medicinal parts. In terms of alkaloid abundance, *E. sinica* Stapf and *E. intermedia* Schrenk et C. A. Mey have higher contents, while MG, XZ, and SL have the second-highest contents. Therefore, these three *Ephedra* varieties can be considered as extension varieties. Additionally, higher altitudes correspond to higher total alkaloid content. In individual alkaloid content comparison, WQM, WQZ, MG, NMG, and XZ have higher levels, indicating greater alkaloid content in regions with higher altitudes. However, higher content is also observed in the Hebei Plain region. For pseudoephedrine content, WQD and WQC exhibit lower levels, corresponding to lower altitudes among the eight *Ephedra* species. For methylephedrine content, higher levels are observed in XZ, NMG, SL, SX, WQC, WQM, and WQZ, indicating greater levels in *Ephedra* from higher altitude areas. Consequently, it is hypothesized that the content of ephedrine, pseudoephedrine, and alkaloids increases with altitude gradient (Kitani et al., 2009; Guo et al., 2022; Lu et al., 2023), as illustrated in **Supplementary Figures S6** and **S7**.

In conclusion, *E. gerardiana* Wall, *E. likiangensis* Florin, *E. przewalskii* Stapf, and *E. saxatilis* Royle ex Florin are recommended for extended medicinal use of Ephedra due to their legal recognition. Although *E. przewalskii* Stapf and *E. monosperma* Gmel. ex Mey do not meet the Pharmacopoeia’s criteria for specified alkaloid content in MHS, their medicinal value remains noteworthy. In recent years, with the expansion of cultivated *Ephedra* and the decrease in the use of wild resources, substitutes for *Ephedra* have not been thoroughly considered. The current study indicates that although they share similar main chemical components, differences exist in the types of components and the relative content of the main active ingredients. Therefore, attention should be paid to potential differences in efficacy. Further research is needed to explore the similarities and differences in their pharmacological activities to ensure the effectiveness and consistency of herbal quality.

## 5 Conclusion

HS-GC-MS and UPLC-Q-TOF-MS techniques were employed to investigate the chemical components of eight *Ephedra* species across different plant parts. The analysis revealed 37 volatile components in MHS and 46 in MHR, including alkenes, terpenoids, aldehydes, and alcohols. Additionally, 42 non-volatile components were identified in both MHS and MHR, including alkaloids, flavonoids, glycosides, carboxylic acids, and fatty acids. The primary differentiating factors between species are alkaloids and flavonoids. Differences between plant parts also involve these compounds, contributing to both similarities and distinctions. Distinctive compounds were further analyzed using biogenic synthesis pathways, including fatty acyl synthesis, phenylpropane synthesis, terpenoid and steroid biosynthesis, lipid synthesis, isoprenoid synthesis, and benzene synthesis. *E. gerardiana* Wall, *E. likiangensis* Florin, *E. przewalskii* Stapf, and *E. saxatilis* Royle ex Florin should be considered as supplements to medicinal *Ephedra* resources. This study provides insights for verifying and safely applying *Ephedra* herbs through differential analysis and comparison of various *Ephedra* species and plant parts. It also provides a scientific foundation for further exploring the medicinal value and resource utilization of *Ephedra* herbs.

## Data availability statement

The original contributions presented in the study are included in the article/**Supplementary Material**. Further inquiries can be directed to the corresponding authors.

## Author contributions

DZ: Conceptualization, Funding acquisition, Project administration, Supervision, Writing – review & editing. BG: Investigation, Methodology, Validation, Writing – original draft, Writing – review & editing. LY: Investigation, Methodology, Validation, Writing – original draft, Writing – review & editing. HL: Data curation, Formal analysis, Methodology, Resources, Writing – review & editing. QA: Data curation, Formal analysis, Methodology, Resources, Writing – review & editing. YL: Data curation, Formal analysis, Methodology, Resources, Writing – review & editing. JC: Data curation, Formal analysis, Methodology, Resources, Writing – review & editing. FH: Conceptualization, Funding acquisition, Project administration, Resources, Writing – review & editing. LG: Conceptualization, Funding acquisition, Project administration, Supervision, Writing – review & editing.
