# Supplementary material for: Exploration of chemical components and metabolite synthesis pathways in eight Ephedra species based on HS-GC-MS and UPLC-Q-TOF-MS
Source: Front Plant Sci. 2024 Jun 12;15:1421008. doi: 10.3389/fpls.2024.1421008 (PMC11205265; doi:10.3389/fpls.2024.1421008)
Supplement: Supplementary file 1 [file DataSheet_1.docx]

Supplementary Materials

Exploration of Chemical Components and Metabolite Synthesis Pathways in *Ephedra* species Based on HS-GC-MS and UPLC-Q-TOF-MS

Bing Guo^a,b,1^, Lina Yang^a,b,1^, Hengyang Li^a,b^, Qi An^c^, Yongli Liu^c^, Jie Cheng^d^, Fangjie Hou^a,b*^, Long Guo^a,b*^, Dan Zhang^a,b*^

*a. Traditional Chinese Medicine Processing Technology Innovation Centre of Hebei Province,* *College of Pharmacy,* *Hebei University of Chinese Medicine, Shijiazhuang 050200, China;*

*b. International Joint Research Centre on Resource Utilization and Quality Evaluation of Traditional Chinese Medicine of Hebei Province, Shijiazhuang, 050200, China;*

*c. Department of Chinese Materia Medica, Hebei Institute for Drug and Medical Device Control, Shijiazhuang, 050200, China*

*d. Hebei Industrial Technology Institute for Traditional Chinese Medicine Preparation, The First Affiliated Hospital of Hebei University of Chinese Medicine, Shijiazhuang 050200, China;*

^1^ *These authors contributed equally to this work.*

* *Corresponding authors.* Tel.: +86 311-89936466, Fax: +86 311-89926000

*E-mail address:* zhangdan@hebcm.edu.cn (D. Zhang*), guo_long11@163.com (L. Guo*), 15130687505@163.com (F.-J. Hou)

**1 Supplementary Figures and Tables**

**1.1 Supplementary Figures**

**Fig. S1.** The diagram of *Ephedra* stems and roots

**Fig. S2.** Possible cleavage patterns of Norephedrine, *l*-norpseudoephedrine, ephedrine, eseudoephedrine, eethylephedrine

**Fig. S3.** Possible cleavage patterns of ephedradine B/D and ephedradine A

**Fig. S4.** Possible cleavage patterns of catechin

**Fig. S5.** Synthetic pathways of the main chemical components of *Ephedra* stem and root

**Fig. S6.** Determination of three kinds of total alkaloids in *Ephedra* stems

**Fig. S7.** Determination of three alkaloids in *Ephedra* stems

**Fig. S8.** Structures of 42 components in MHS identified by HPLC-Q-TOF-MS.

**Fig. S9.** Structures of 42 components in MHR identified by HPLC-Q-TOF-MS.

**Fig. S1.** The diagram of *Ephedra* stems and roots


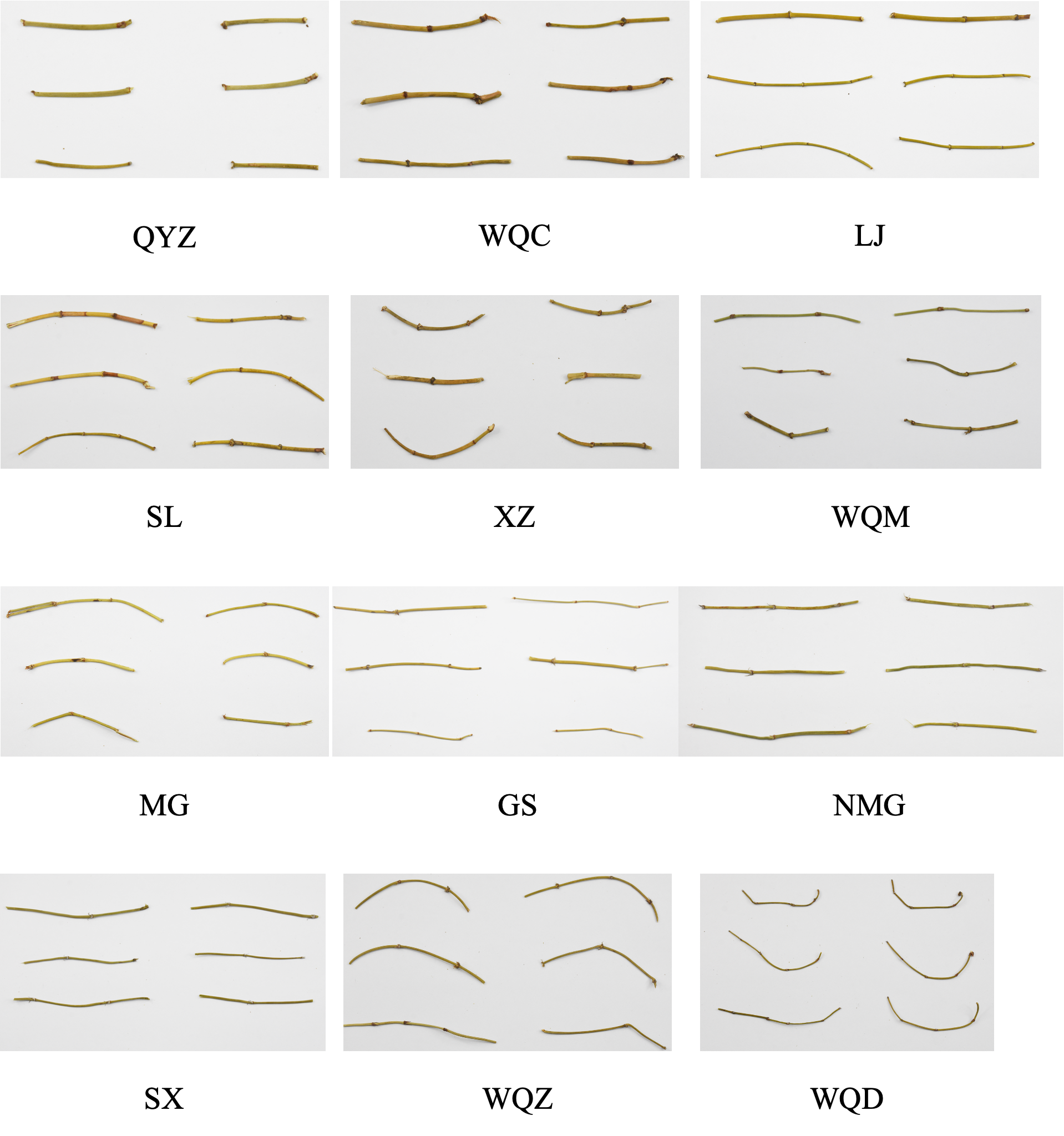


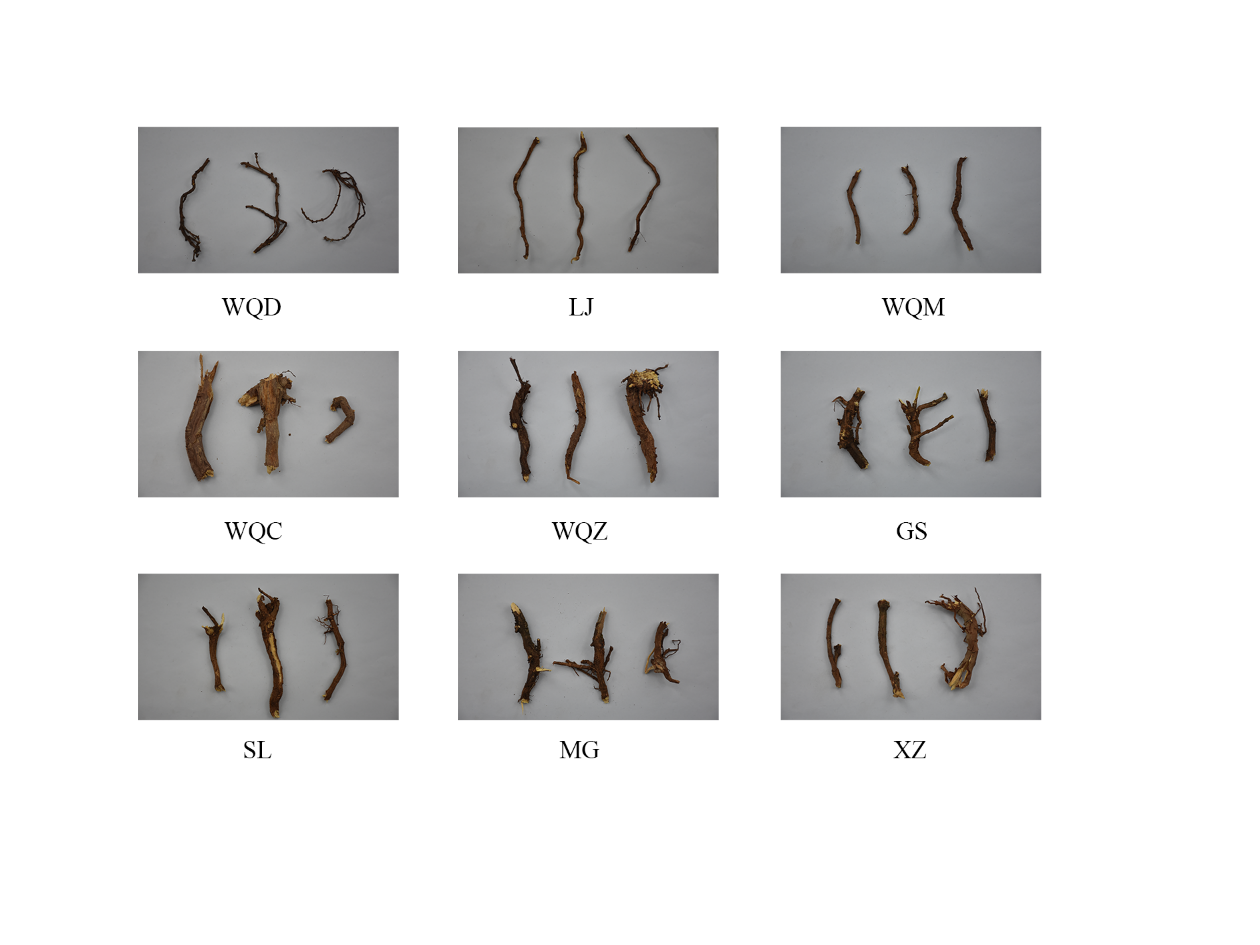


**Fig. S2.** Possible cleavage patterns of norephedrine, *l*-norpseudoephedrine, ephedrine, pseudoephedrine, methylephedrine


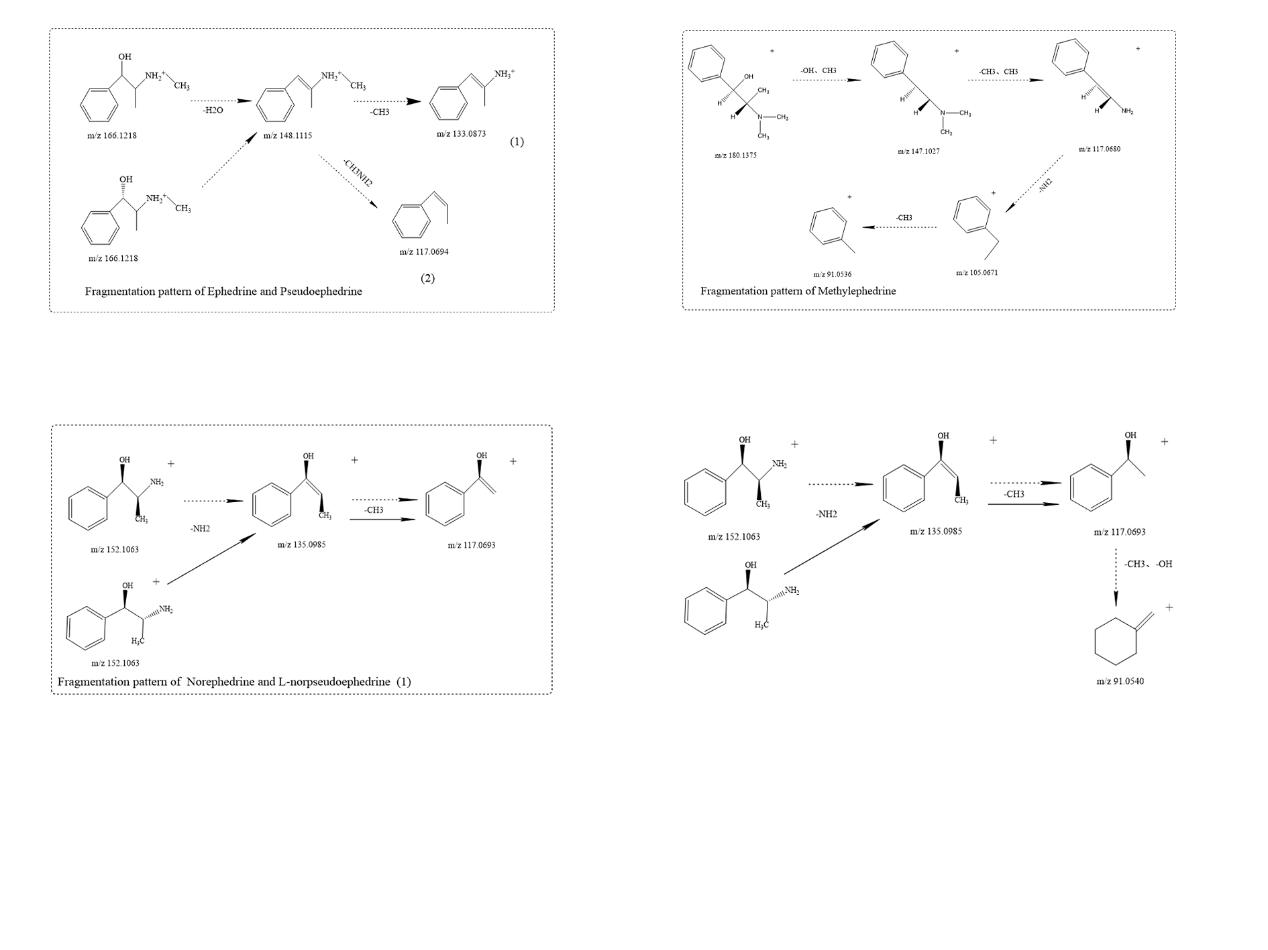


**Fig. S3.** Possible cleavage patterns of ephedradine B/Dand ephedradine A


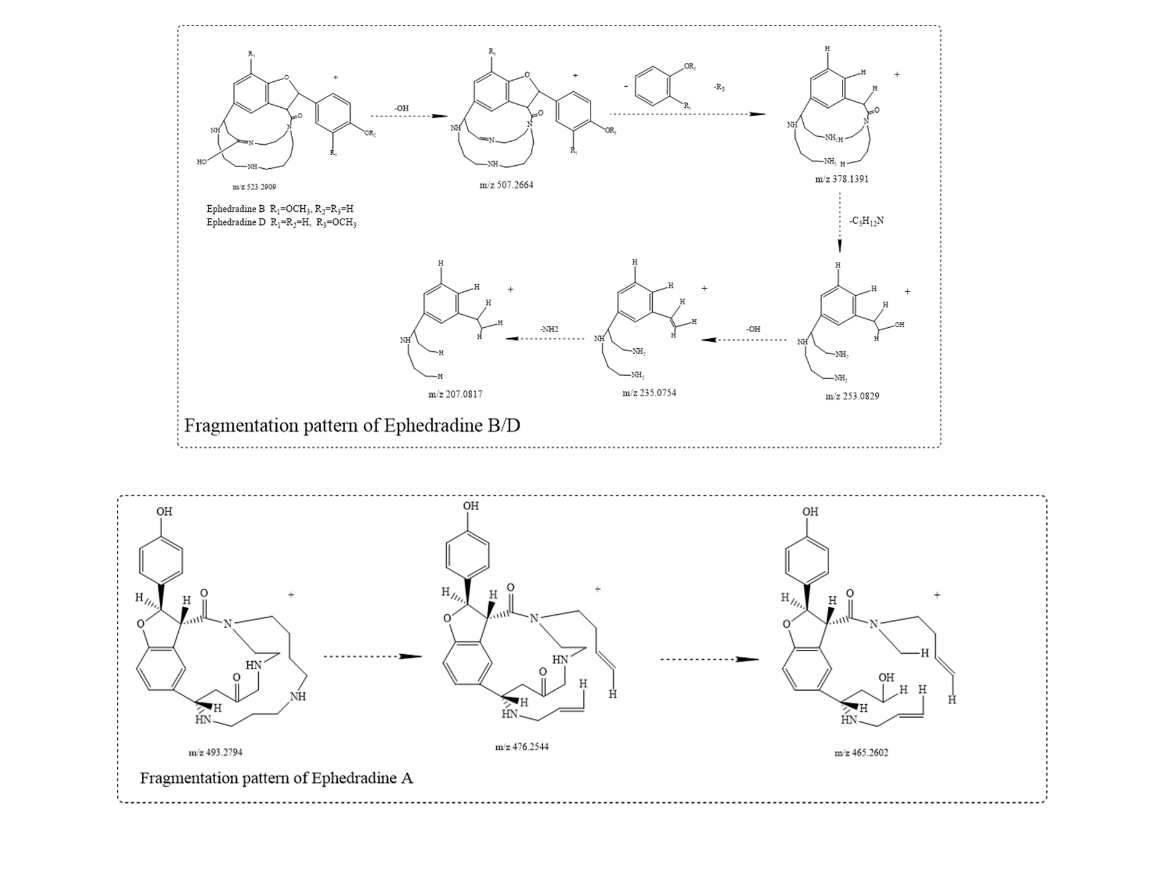


**Fig. S4.** Possible cleavage patterns of catechin


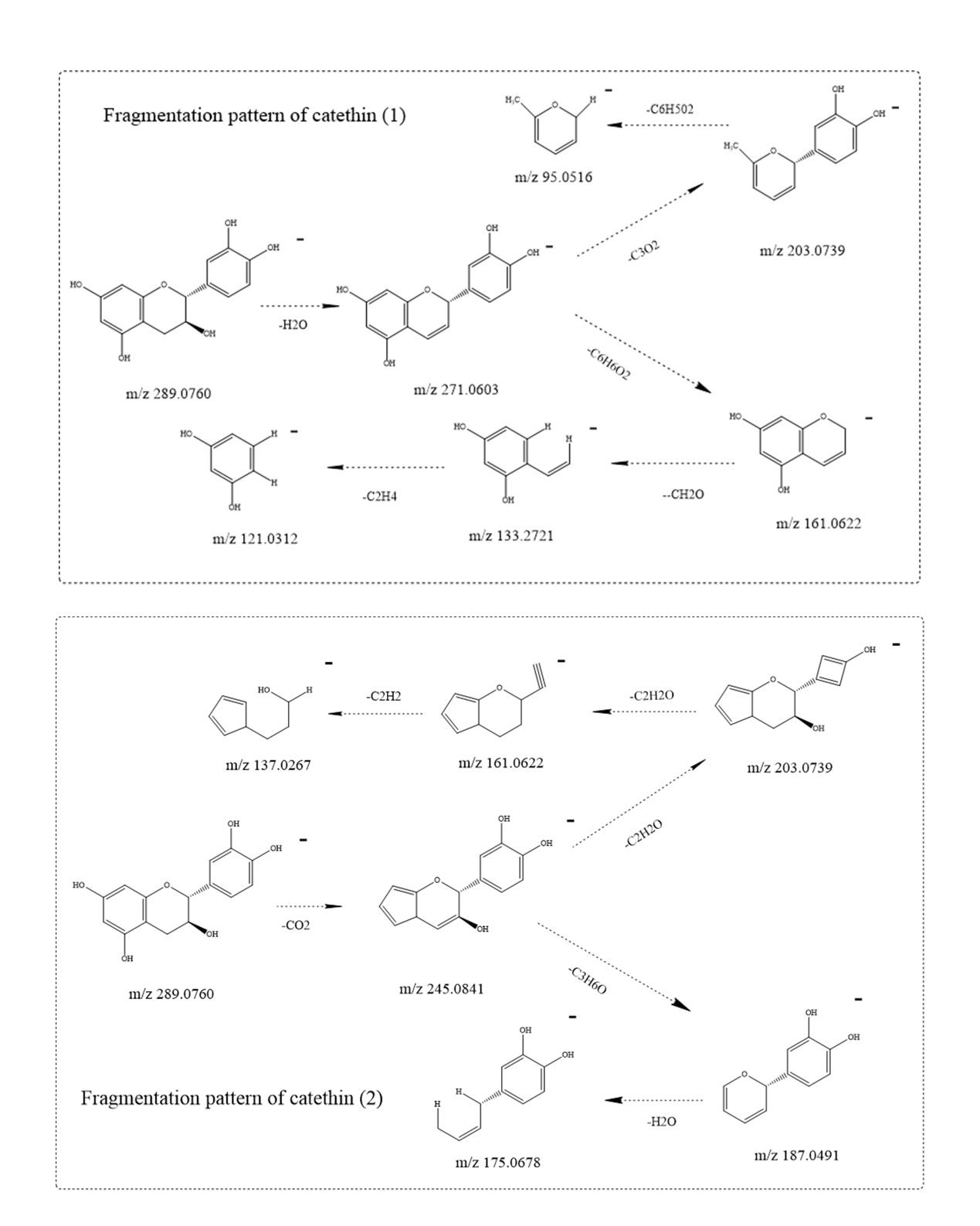


**Fig. S5.** Synthetic pathways of the main chemical components of *Ephedra* stem and root


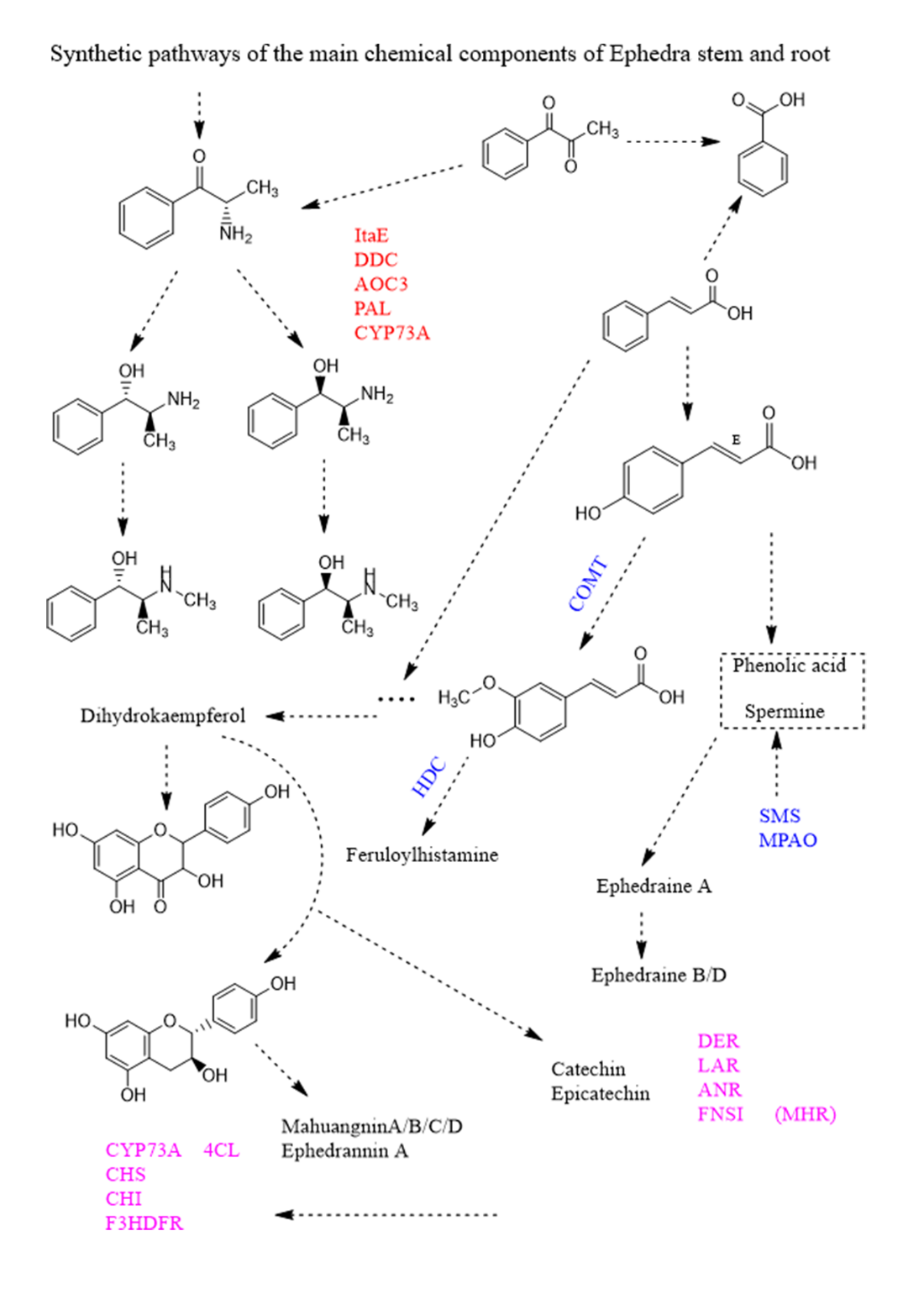


**Fig. S6.** Determination of three kinds of total alkaloids in *Ephedra* stems


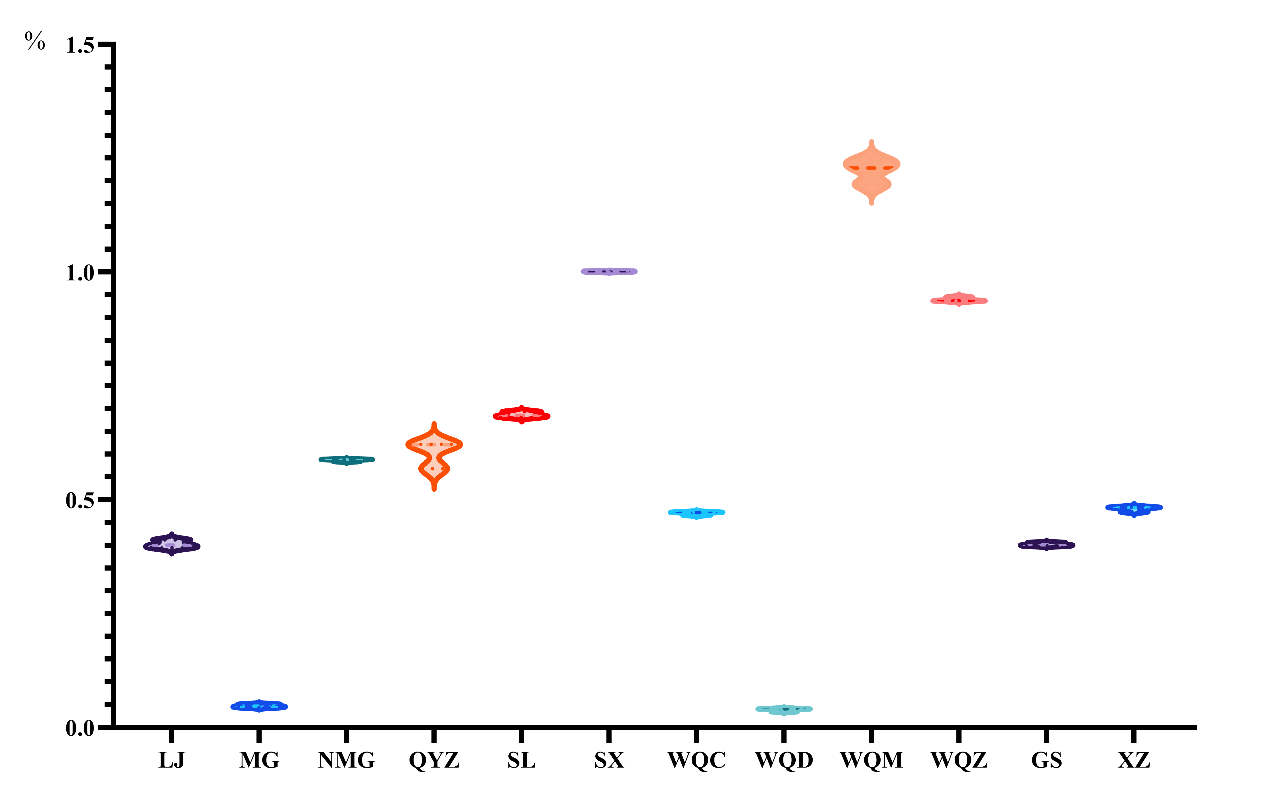


**Fig. S7.** Determination of three alkaloids in *Ephedra* stems


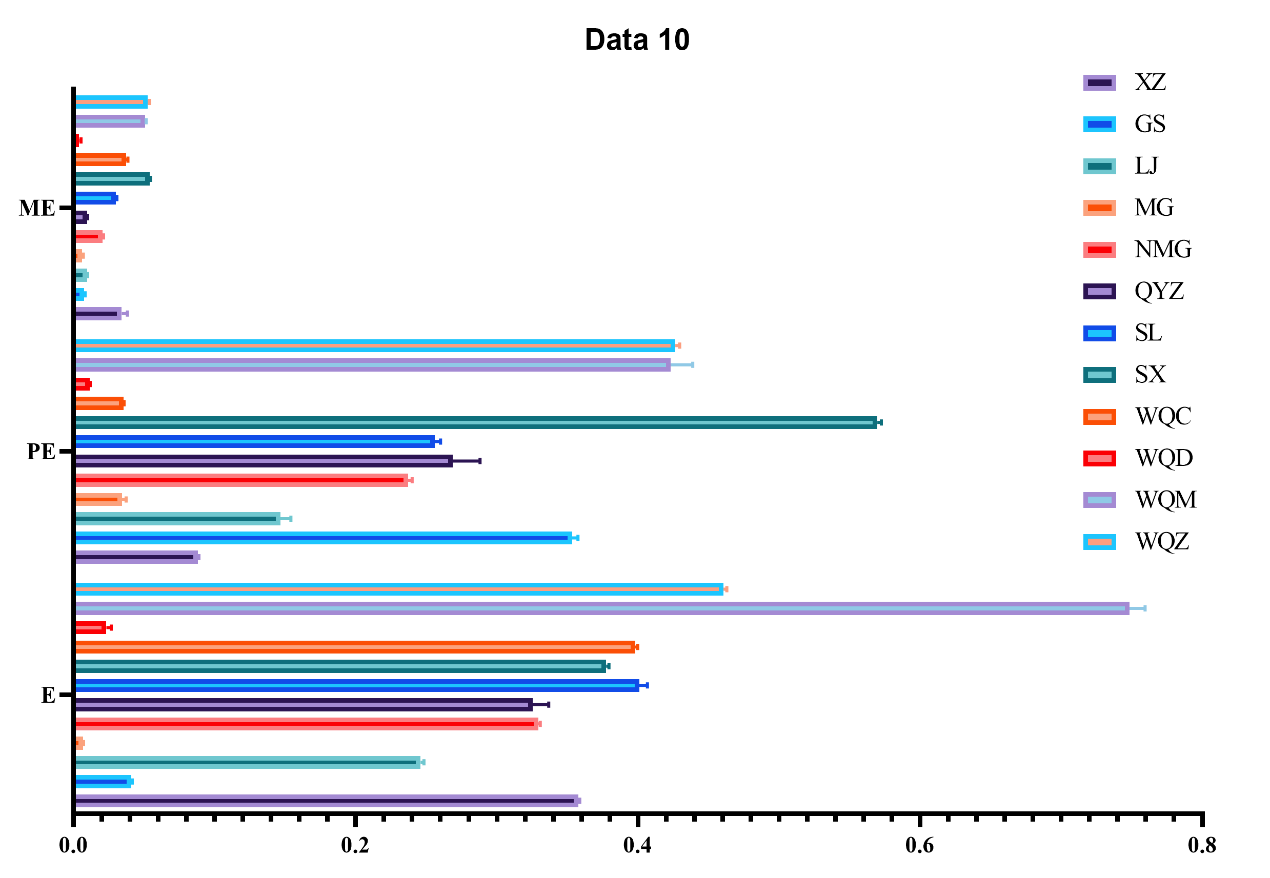


**Fig. S8.** Structures of 42 components in MHS identified by HPLC-Q-TOF-MS.


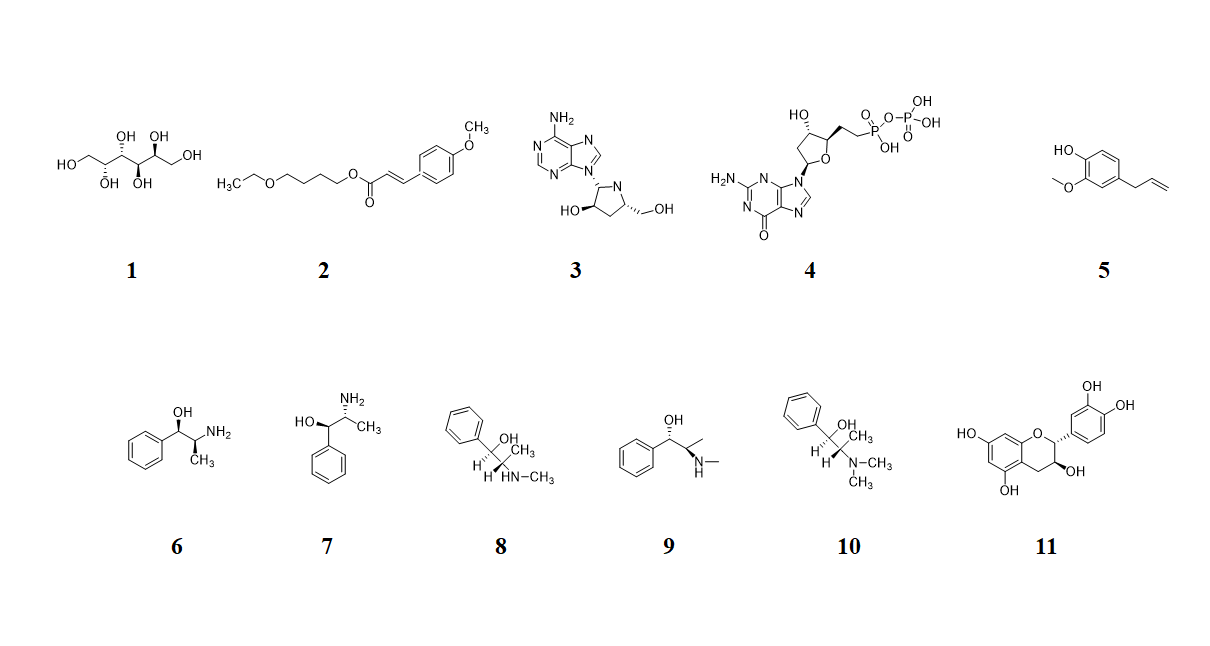


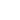

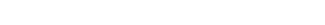

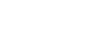

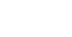

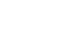

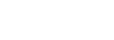

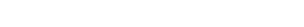

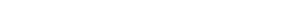

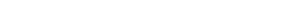


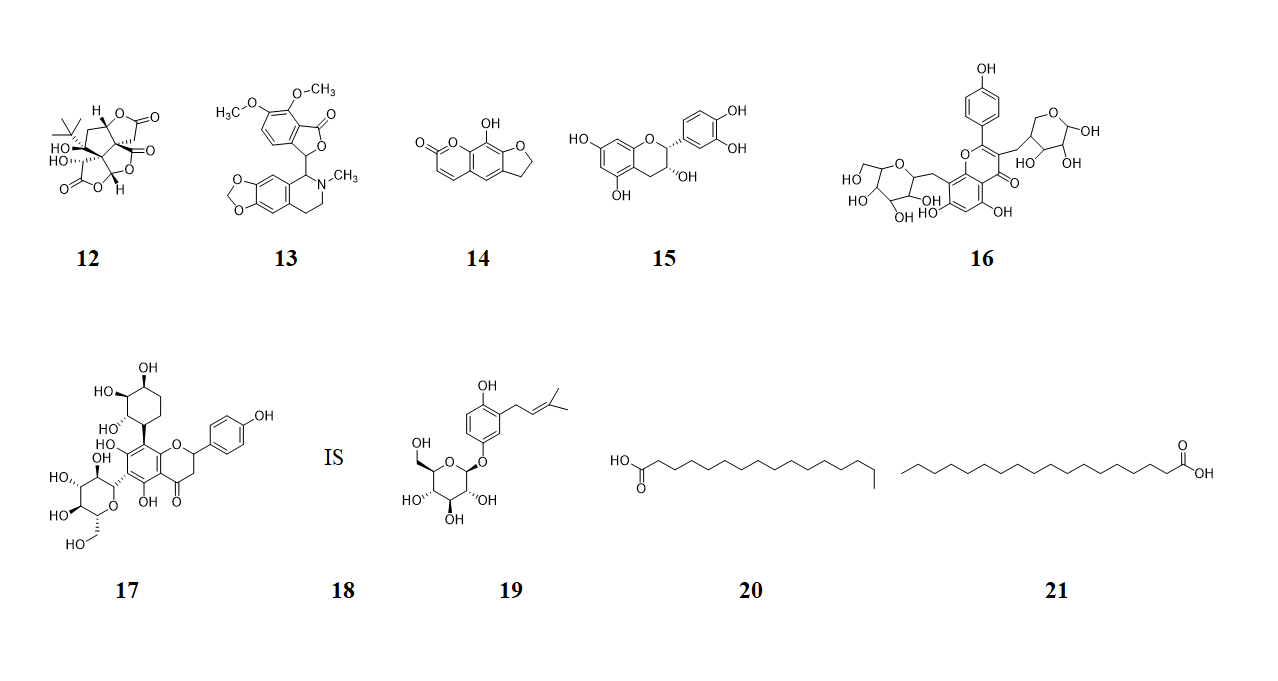


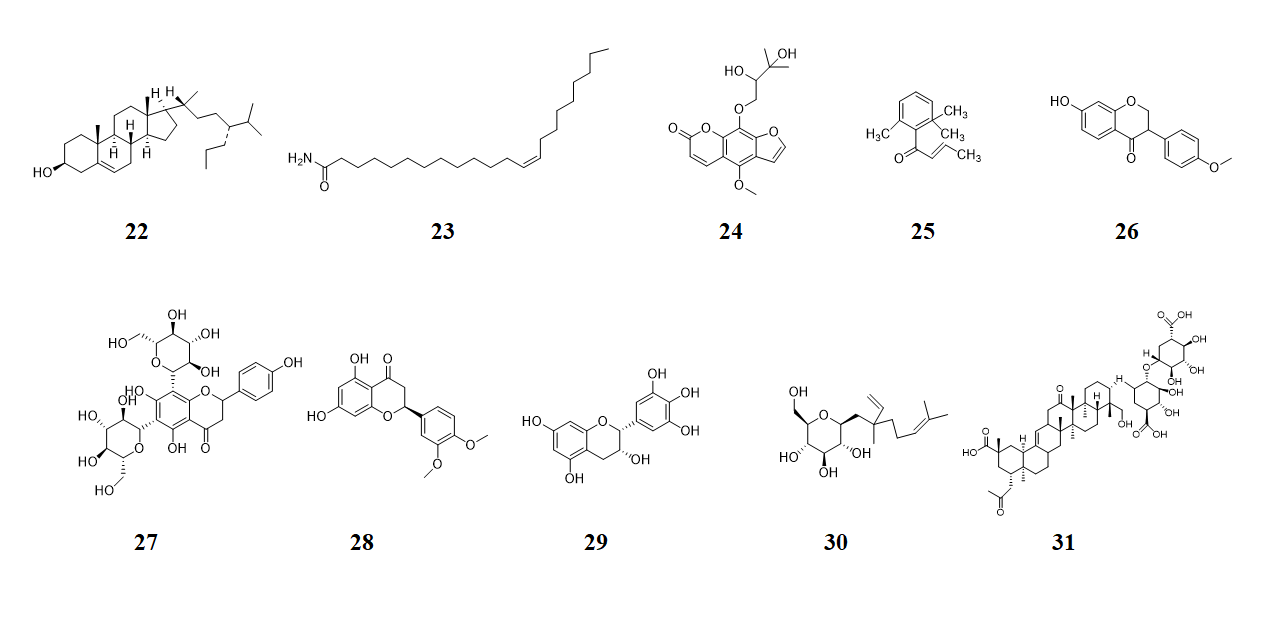


**
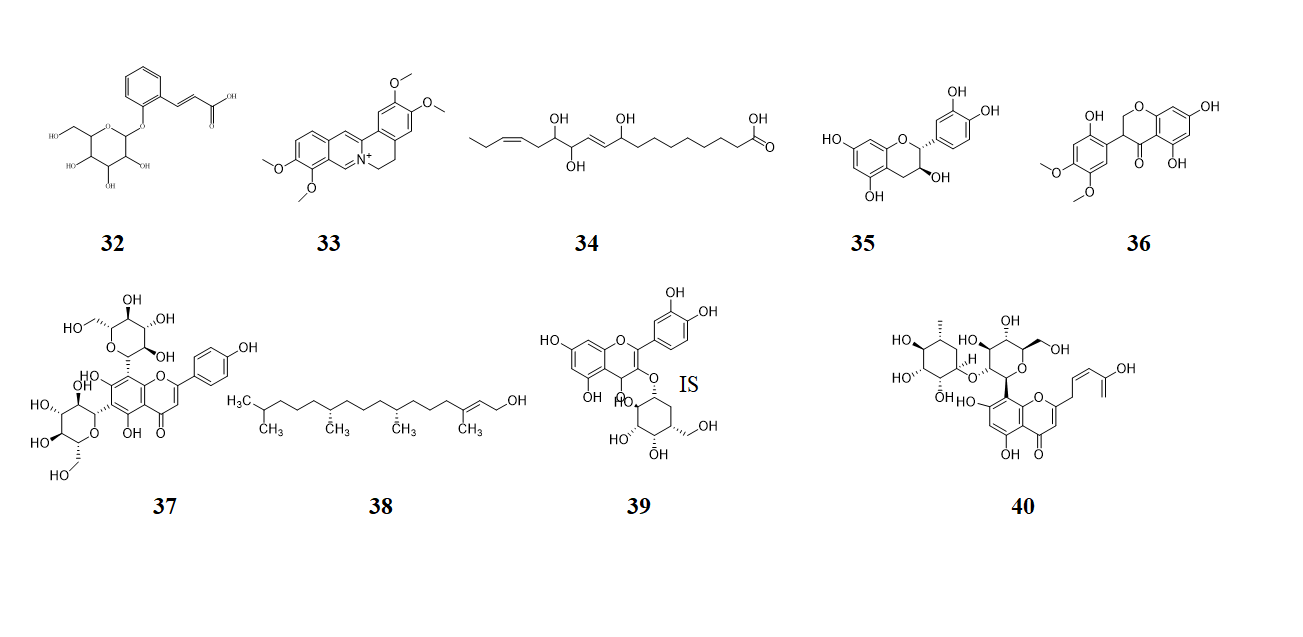
**

**
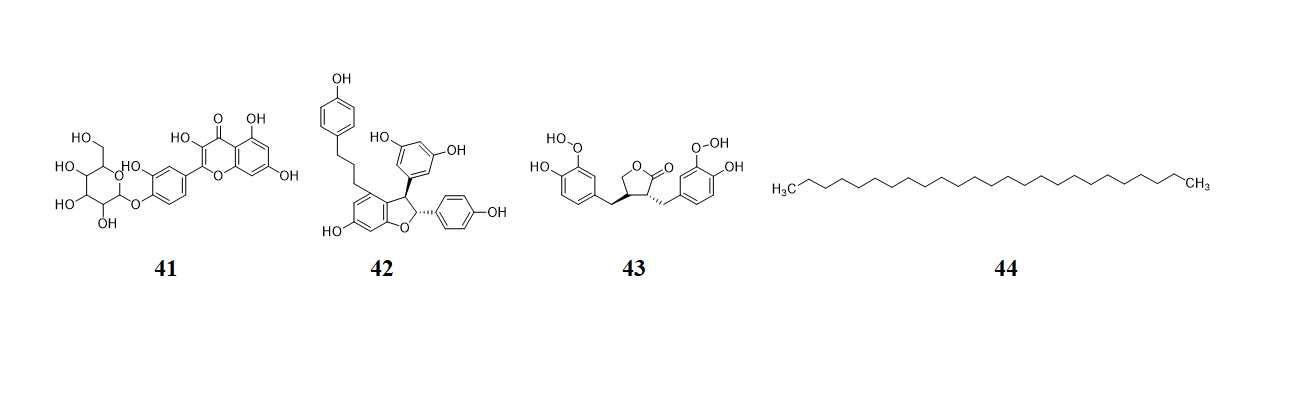
**

**Fig. S9.** Structures of 42 components in MHR identified by HPLC-Q-TOF-MS.


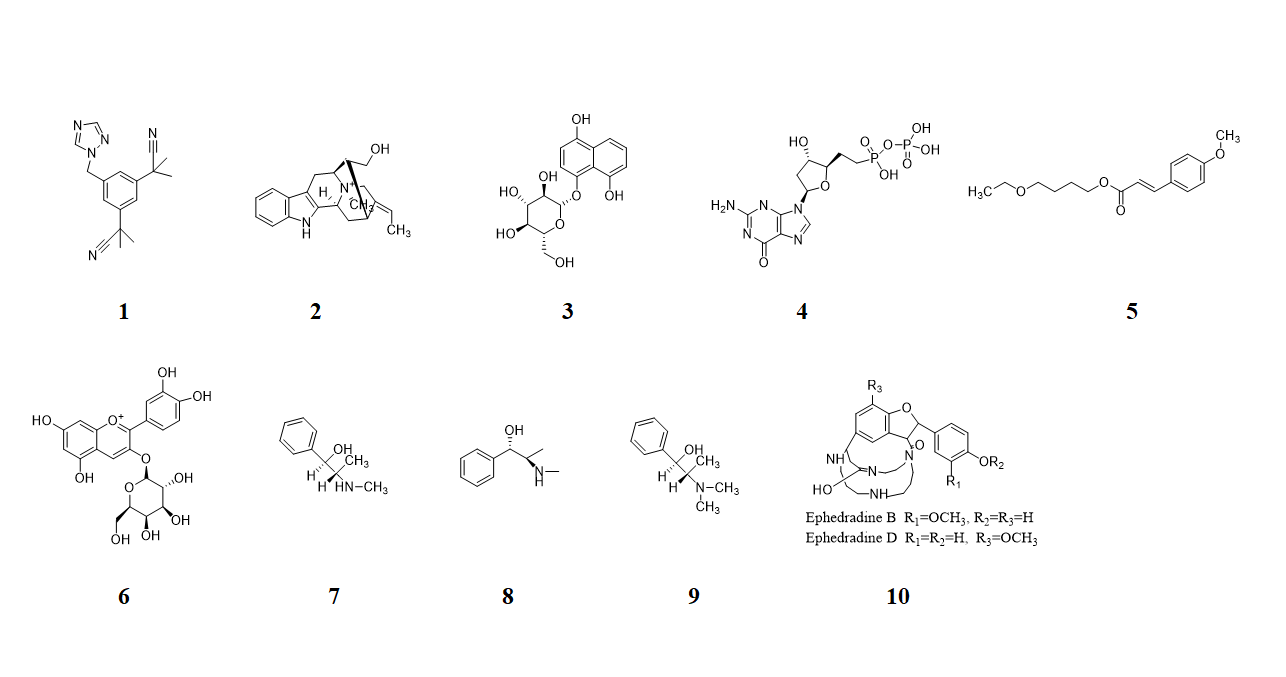


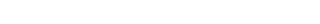

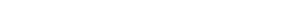

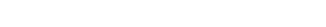

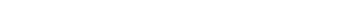

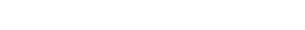


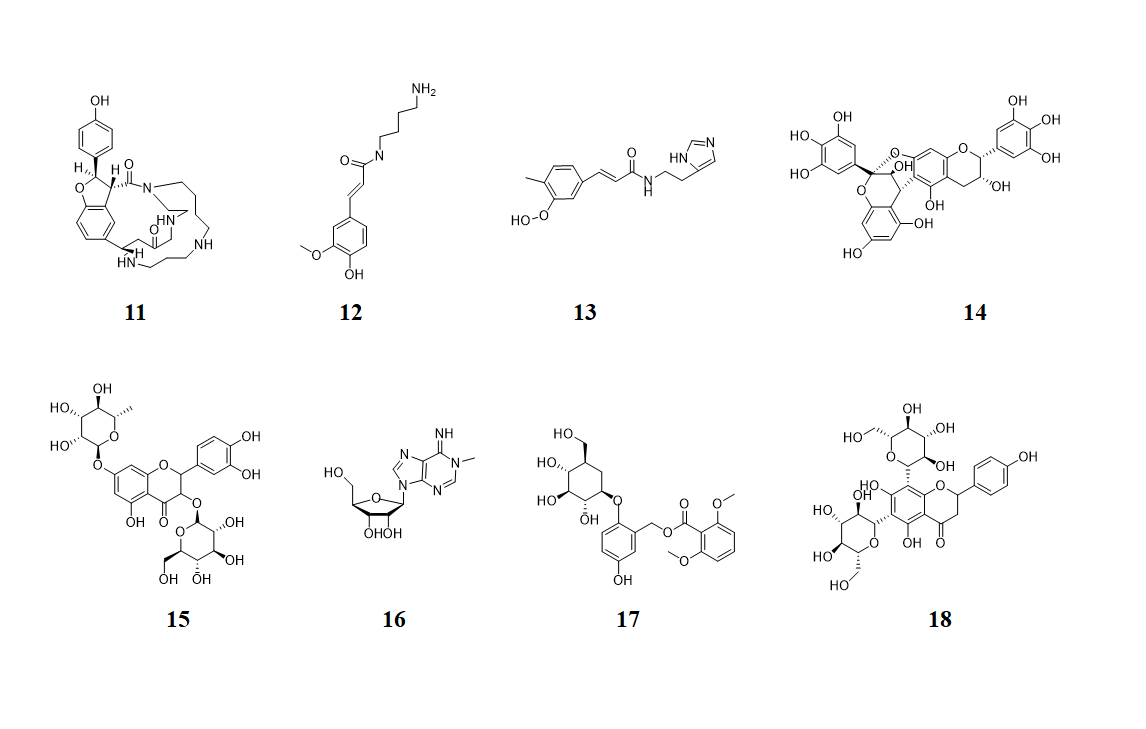


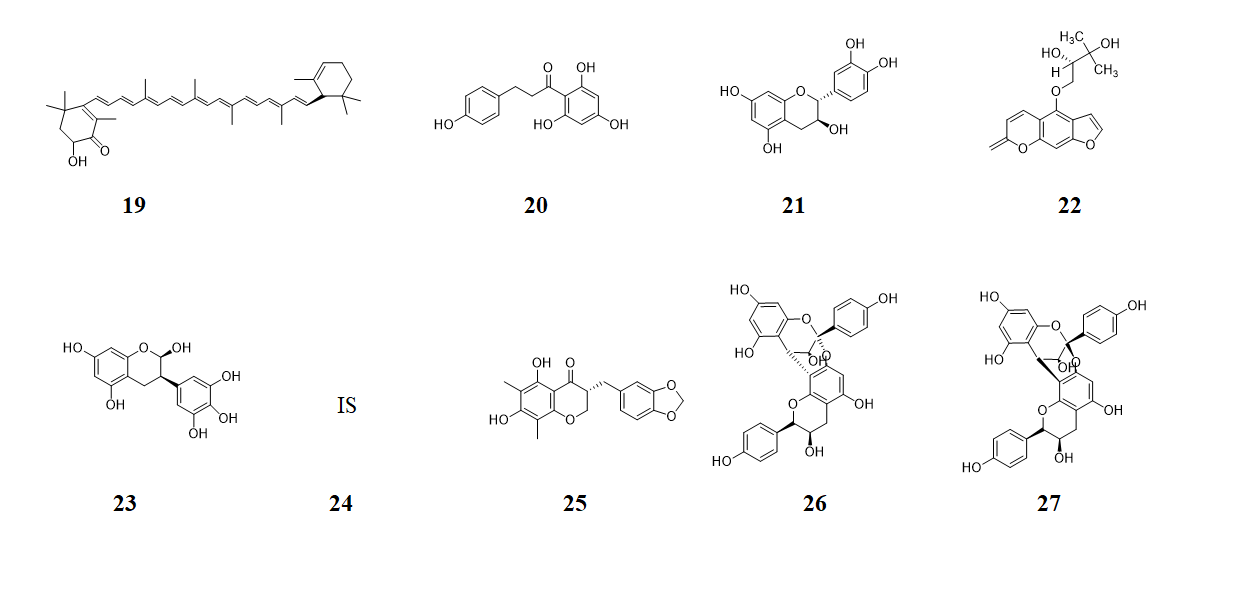

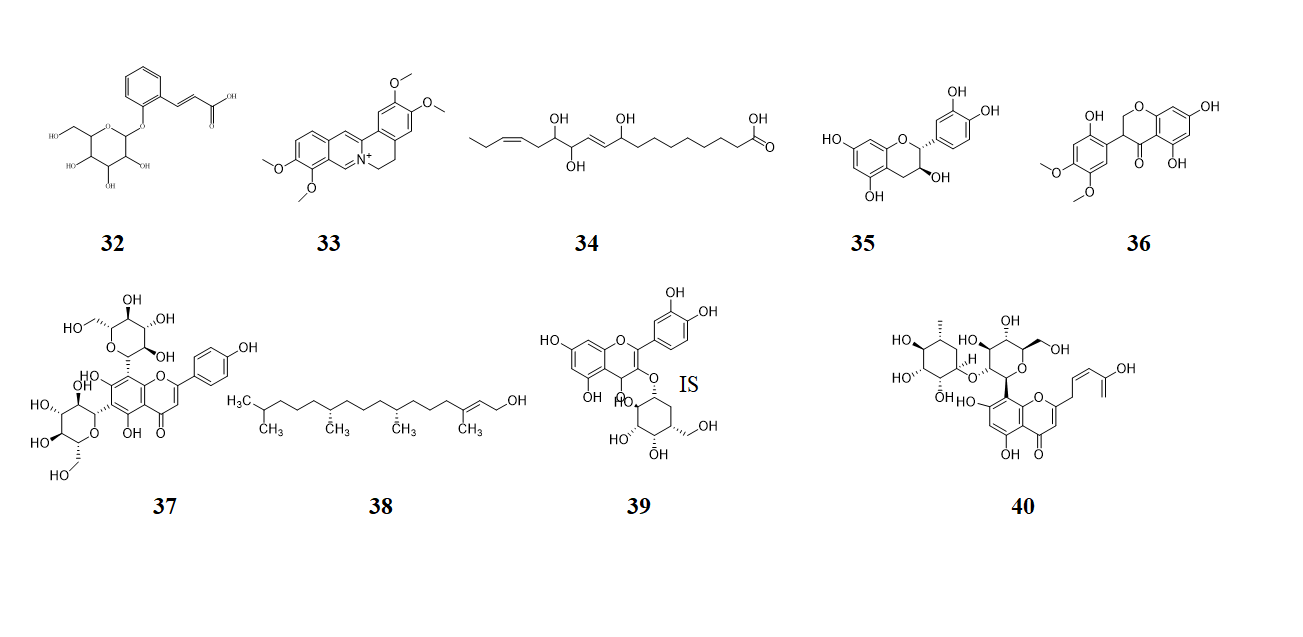

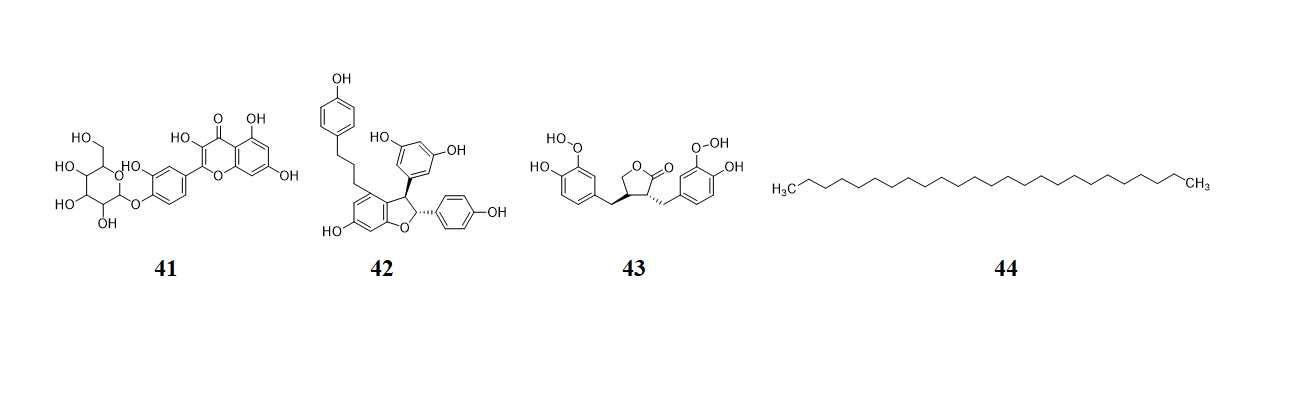


**1.2 Supplementary Tables**

**Table S1** Differential Volatile component VIP values of MHS and MHR

**Table S2** Relative content of differential component in MHS

**Table S3** Relative content of differential component in MHR

**Table S4** Differential non-volatile component VIP values of MHS and MHR

**Table S1** Differential Volatile component VIP values of MHS and MHR

| MHS | VIP | MHR | VIP |
| --- | --- | --- | --- |
| Alloaromadendrene | 1.28374 | 2,3-Butanediol | 1.31732 |
| Heptanal | 1.27275 | Furfural | 1.1937 |
| 1,6-Dimethylhepta-1,3,5-triene | 1.26948 | Cyclopentanecarboxylic acid, 1-methyl- | 1.19083 |
| 1-ethenyl-4-methoxy-Benzene | 1.16429 | 1-Hexanol | 1.16421 |
| 3-methyl-1-Pentanol | 1.16282 | 4-Cyclopentene-1,3-diol, *cis*- | 1.16071 |
| (2*S*,4*R*)-4-Methyl-2-(2-methylprop-1-en-1-yl)tetrahydro-2*H*-pyran | 1.15423 | Heptanal | 1.15726 |
| 6-methyl-2-Heptanone | 1.12385 | Bicyclo[3.1.1]hept-2-ene, 3,6,6-trimethyl- | 1.15093 |
| 1-phenyl-1,2-Propanedione | 1.11202 | 2-Heptanone, 6-methyl- | 1.12938 |
| 2-ethenyltetrahydro-2,6,6-trimethyl-2*H*-Pyran | 1.07591 | Benzaldehyde | 1.12153 |
| Styrene | 1.06411 | 2-Furancarboxaldehyde, 5-methyl- | 1.09573 |
| Ethyl2-(5-methyl-5-vinyltetrahydrofuran-2-yl)propan-2-yl carbona | 1.04974 | Bicyclo[3.1.1]heptane, 6,6-dimethyl-2-methylene-, (1*S*)- | 1.09469 |
| 2-Methyl-1-octen-3-yne | 1.04849 | 1-Octen-3-ol | 1.07622 |
| Benzaldehyde | 1.03963 | 5-Hepten-2-one, 6-methyl- | 1.07511 |
| *α*-Bisabolol | 1.03729 | Hexanoic acid | 1.07303 |
| 2.3-dimethyl- Bicyclo[2.2.1]hept-2-ene. | 1.02681 | Benzene, (2-methyl-1-propenyl)- | 1.05235 |
| tetrahydro-2,2-dimethyl-5-(1-methyl-1-propenyl)-Furan | 1.02058 | Nonanal | 1.04547 |
| 3.4-Dimethylcyclohexanol | 1.00871 | Cyclohexanone, 5-methyl-2-(1-methylethyl)-, t*rans*- | 1.0337 |
| *cis*-*α*-Bergamotene | 1.00146 | 2-Methoxy-5-methylphenol | 1.03367 |
|  |  | Benzene, 1-methoxy-4-methyl-2-(1-methylethyl)- | 1.03321 |
|  |  | Carvone | 1.03257 |
|  |  | (*E*)-*β*-Famesene | 1.02202 |

**Table S2** Relative content of differential component in MHS

| Name | LJ | MG | NMG | QYZ | SL | SX | WQC | WQD | WQM | WQZ | GS | XZ |
| --- | --- | --- | --- | --- | --- | --- | --- | --- | --- | --- | --- | --- |
| 1,6-Dimethylhepta-1,3,5-triene | 0.034±0.001 | 0.029±0.003 | 0.022±0.001 | 0.035±0.002 | 0.022±0.001 | 0.026±0.001 | 0.024±0.002 | 0.024±0.001 | 0.03±0.001 | 0.038±0.003 | 0.025±0.002 | 0.024±0.003 |
| 3-methyl-1-Pentanol | 0.026±0.007 | 0.037±0.006 | 0.047±0.003 | 0.019±0.001 | 0.022±0.003 | 0.035±0.004 | 0.021±0.002 | 0.028±0.005 | 0.014±0.001 | 0.019±0.002 | 0.032±0.003 | 0.033±0.002 |
| 2,3-dimethyl- Bicyclo[2.2.1]hept-2-ene | 0.022±0.000 | 0.016±0.002 | 0.013±0.002 | 0.03±0.001 | 0.021±0.011 | 0.012±0.001 | 0.011±0.001 | 0.011±0.001 | 0.089±0.006 | 0.018±0.001 | 0.018±0.002 | 0.071±0.01 |
| Styrene | 0.008±0.000 | 0.086±0.018 | 0.039±0.006 | 0.016±0.001 | 0.049±0.005 | 0.018±0.001 | 0.06±0.019 | 0 | 0.018±0.001 | 0.01±0.001 | 0.006±0.001 | 0.023±0.001 |
| Heptanal | 0.09±0.01 | 0.021±0.001 | 0.026±0.002 | 0.014±0.001 | 0.019±0.002 | 0.016±0.001 | 0.021±0.001 | 0.021±0.001 | 0.012±0.001 | 0.021±0.002 | 0.05±0.004 | 0.021±0.005 |
| 6-methyl-2-Heptanone | 0.023±0.005 | 0.05±0.019 | 0.035±0.003 | 0.025±0.001 | 0.022±0.003 | 0.011±0.001 | 0.02±0.006 | 0.007±0.000 | 0.05±0.003 | 0.009±0.001 | 0.043±0.005 | 0.039±0.004 |
| Benzaldehyde | 0.034±0.008 | 0.021±0.003 | 0.027±0.001 | 0.025±0.002 | 0.03±0.001 | 0.037±0.003 | 0.021±0.00 | 0.006±0.000 | 0.026±0.001 | 0.029±0.001 | 0.046±0.006 | 0.033±0.004 |
| 2-ethenyltetrahydro-2,6,6-trimethyl-2*H*-Pyran, | 0 | 0.133±0.053 | 0.048±0.008 | 0 | 0.009±0.003 | 0.102±0.006 | 0.018±0.002 | 0 | 0.005±0.002 | 0.007±0.001 | 0 | 0.011±0.000 |
| 2-Methyl-1-octen-3-yne | 0.002±0.000 | 0.031±0.019 | 0.013±0.002 | 0.006±0.000 | 0.023±0.003 | 0.009±0.001 | 0.009±0.001 | 0.005±0.000 | 0.072±0.004 | 0.027±0.004 | 0.073±0.009 | 0.063±0.009 |
| Tetrahydro-2,2-dimethyl-5-(1-methyl-1-propenyl)-Furan | 0 | 0.118±0.005 | 0.043±0.003 | 0 | 0.013±0.003 | 0.082±0.005 | 0.021±0.004 | 0 | 0.008±0.003 | 0.006±0.000 | 0.005±0.001 | 0.037±0.001 |
| Ethyl 2-(5-methyl-5-vinyltetrahydrofuran-2-yl)propan-2-yl carbonate | 0.005±0.001 | 0.049±0.014 | 0.035±0.005 | 0.002±0.001 | 0.079±0.006 | 0.035±0.002 | 0.017±0.002 | 0 | 0.008±0.001 | 0.007±0.001 | 0.009±0.001 | 0.087±0.007 |
| 3,4-Dimethylcyclohexanol | 0.023±0.002 | 0.038±0.004 | 0.03±0.001 | 0.016±0.002 | 0.023±0.004 | 0.027±0.001 | 0.026±0.002 | 0.021±0.001 | 0.016±0.001 | 0.027±0.004 | 0.039±0.003 | 0.047±0.002 |
| (2*S*,4*R*)-4-Methyl-2-(2-methylprop-1-en-1-yl)tetrahydro-2*H*-pyran | 0.052±0.006 | 0.005±0.001 | 0.007±0.001 | 0.009±0.000 | 0.015±0.001 | 0.114±0.015 | 0.01±0.00 | 0.023±0.002 | 0.007±0.001 | 0.001±0.001 | 0.058±0.018 | 0.032±0.006 |
| 1-ethenyl-4-methoxy-Benzene, | 0 | 0.016±0.003 | 0.044±0.015 | 0.005±0.00 | 0.006±0.002 | 0.052±0.001 | 0.049±0.012 | 0 | 0.118±0.002 | 0.016±0.004 | 0.015±0.002 | 0.013±0.003 |
| 1-phenyl-1,2-Propanedione | 0.054±0.025 | 0.006±0.002 | 0.016±0.006 | 0.06±0.004 | 0.034±0.002 | 0.018±0.002 | 0.023±0.001 | 0 | 0.015±0.001 | 0.014±0.002 | 0.091±0.007 | 0 |
| *α*-Bisabolol | 0.048±0.007 | 0.023±0.002 | 0.024±0.002 | 0.017±0.003 | 0.07±0.025 | 0.028±0.002 | 0.01±0.001 | 0.003±0.001 | 0.009±0.001 | 0.012±0.001 | 0.07±0.031 | 0.022±0.001 |
| *Cis*-*α*-Bergamotene | 0.049±0.008 | 0.024±0.003 | 0.018±0.001 | 0.015±0.002 | 0.073±0.025 | 0.02±0.001 | 0.011±0.004 | 0.002±0.002 | 0.008±0.002 | 0.009±0.001 | 0.077±0.041 | 0.027±0.006 |
| Alloaromadendrene | 0.027±0.009 | 0.018±0.004 | 0.038±0.003 | 0.06±0.003 | 0.017±0.003 | 0.058±0.009 | 0.029±0.002 | 0.003±0.001 | 0.024±0.002 | 0.015±0.003 | 0.015±0.003 | 0.03±0.005 |

**Table S3** Relative content of differential component in MHR

| Name | LJ | MG | SL | WQZ | XZ | WQM | WQD | WQC | GS |
| --- | --- | --- | --- | --- | --- | --- | --- | --- | --- |
| 2,3-Butanediol | 0.016 ±0.003 | 0.103 ±0.009 | 0.030 ±0.002 | 0.006 ±0.001 | 0.079 ±0.009 | 0.038 ±0.018 | 0.016 ±0.006 | 0.028 ±0.009 | 0.016 ±0.001 |
| Furfural | 0.041 ±0.003 | 0.015 ±0.004 | 0.034 ±0.002 | 0.037 ±0.003 | 0.046 ±0.011 | 0.025 ±0.002 | 0.036 ±0.007 | 0.028 ±0.002 | 0.073 ±0.021 |
| Cyclopentanecarboxylic acid,1-methyl- | 0.120 ±0.052 | 0.002 ±0.001 | 0.080 ±0.023 | 0.009 ±0.000 | 0.036 ±0.003 | 0.027 ±0.022 | 0.027 ±0.009 | 0.015 ±0.006 | 0.018 ±0.004 |
| 1-Hexanol | 0.033 ±0.004 | 0.044 ±0.002 | 0.033 ±0.004 | 0.037 ±0.001 | 0.044 ±0.002 | 0.034 ±0.004 | 0.053 ±0.004 | 0.028 ±0.002 | 0.028 ±0.004 |
| 4-Cyclopentene-1,3-diol,*cis*- | 0.113 ±0.034 | 0.002 ±0.000 | 0.072 ±0.016 | 0.015 ±0.001 | 0.046 ±0.003 | 0.022 ±0.015 | 0.030 ±0.008 | 0.014 ±0.005 | 0.021 ±0.004 |
| Heptanal | 0.069 ±0.003 | 0.020 ±0.005 | 0.050 ±0.005 | 0.037 ±0.002 | 0.032 ±0.004 | 0.029 ±0.003 | 0.034 ±0.004 | 0.030 ±0.003 | 0.031 ±0.002 |
| Bicyclo[3.1.1]hept-2-ene,3,6,6-trimethyl- | 0.036 ±0.015 | 0.037 ±0.004 | 0.032 ±0.004 | 0.006 ±0.003 | 0.031 ±0.002 | 0.039 ±0.006 | 0.048 ±0.001 | 0.077 ±0.038 | 0.026 ±0.004 |
| 2-Heptanone,6-methyl- | 0.026 ±0.001 | 0.033 ±0.002 | 0.033 ±0.001 | 0.079 ±0.006 | 0.063 ±0.003 | 0.028 ±0.005 | 0.025 ±0.001 | 0.020 ±0.004 | 0.026 ±0.001 |
| Benzaldehyde | 0.041 ±0.002 | 0.031 ±0.003 | 0.040 ±0.002 | 0.050 ±0.005 | 0.045 ±0.002 | 0.033 ±0.007 | 0.027 ±0.002 | 0.032 ±0.000 | 0.034 ±0.003 |
| 2-Furancarboxaldehyde,5-methyl- | 0.023 ±0.002 | 0.017 ±0.004 | 0.028 ±0.010 | 0.018 ±0.003 | 0.041 ±0.024 | 0.020 ±0.004 | 0.021 ±0.001 | 0.024 ±0.006 | 0.141 ±0.060 |
| Bicyclo[3.1.1]heptane, 6,6-dimethyl-2-methylene-,(1*S*)- | 0.077 ±0.048 | 0.011 ±0.000 | 0.033 ±0.006 | 0.004 ±0.001 | 0.022 ±0.000 | 0.028 ±0.007 | 0.019 ±0.002 | 0.129 ±0.097 | 0.010 ±0.001 |
| 1-Octen-3-ol | 0.028 ±0.003 | 0.030 ±0.003 | 0.026 ±0.005 | 0.029 ±0.004 | 0.052 ±0.002 | 0.044 ±0.003 | 0.033 ±0.005 | 0.034 ±0.001 | 0.057 ±0.004 |
| 5-Hepten-2-one,6-methyl- | 0.033 ±0.003 | 0.035 ±0.003 | 0.028 ±0.007 | 0.033 ±0.006 | 0.042 ±0.006 | 0.045 ±0.008 | 0.032 ±0.002 | 0.039 ±0.008 | 0.046 ±0.007 |
| Hexanoic acid | 0.054 ±0.009 | 0.037 ±0.003 | 0.043 ±0.004 | 0.032 ±0.003 | 0.032 ±0.001 | 0.026 ±0.002 | 0.029 ±0.001 | 0.042 ±0.003 | 0.040 ±0.010 |
| Benzene, (2-methyl-1-propenyl)- | 0.026 ±0.004 | 0.026 ±0.001 | 0.025 ±0.005 | 0.022 ±0.003 | 0.042 ±0.004 | 0.050 ±0.008 | 0.057 ±0.017 | 0.043 ±0.008 | 0.043 ±0.008 |
| Nonanal | 0.038 ±0.000 | 0.017 ±0.006 | 0.032 ±0.004 | 0.070 ±0.013 | 0.044 ±0.002 | 0.031 ±0.001 | 0.031 ±0.005 | 0.033 ±0.003 | 0.038 ±0.000 |
| Cyclohexanone,5-methyl-2-(1-methylethyl)-, trans- | 0.040 ±0.003 | 0.020 ±0.002 | 0.028 ±0.001 | 0.039 ±0.003 | 0.033 ±0.001 | 0.048 ±0.006 | 0.061 ±0.006 | 0.037 ±0.001 | 0.026 ±0.001 |
| 2-Methoxy-5-methylphenol | 0.083 ±0.013 | 0.040 ±0.022 | 0.089 ±0.005 | 0.023 ±0.004 | 0.012 ±0.003 | 0.021 ±0.010 | 0.004 ±0.001 | 0.051 ±0.008 | 0.011 ±0.003 |
| Benzene,1-methoxy-4-methyl-2-(1-methylethyl)- | 0.028 ±0.003 | 0.037 ±0.001 | 0.039 ±0.008 | 0.013 ±0.004 | 0.034 ±0.002 | 0.052 ±0.011 | 0.046 ±0.001 | 0.045 ±0.008 | 0.039 ±0.004 |
| Carvone | 0.058 ±0.007 | 0.022 ±0.002 | 0.037 ±0.009 | 0.029 ±0.003 | 0.034 ±0.007 | 0.045 ±0.001 | 0.046 ±0.008 | 0.039 ±0.008 | 0.023 ±0.003 |
| (*E*)-*β*-Famesene | 0.029 ±0.002 | 0.034 ±0.006 | 0.036 ±0.012 | 0.022 ±0.005 | 0.036 ±0.006 | 0.050 ±0.008 | 0.037 ±0.005 | 0.044 ±0.001 | 0.048 ±0.003 |

**Table S4** Differential non-volatile component VIP values of MHS and MHR

| MHS | VIP | MHR | VIP |
| --- | --- | --- | --- |
| Norephedrine | 1.10636 | Austinoneol | 1.24677 |
| Dehydrocorydaline | 1.09651 | Ephedradine A | 1.12308 |
| Beta-Sitosterol | 1.09588 | Kaempferol-3-*O*-glucoside-7-*O*-rhamnoside | 1.09955 |
| octacosane | 1.09153 | Phloretin | 1.09534 |
| Ethyl-*p*-methoxycinnamate | 1.08769 | 2'-Deoxyguanosine 5'-diphosphate | 1.0743 |
| Formononetin | 1.07271 | Cyanidin-3-*O*-galactoside | 1.06847 |
| (10*E*,15*E*)-9,12,13-trihydroxyoctadeca-10,15-dienoic acid | 1.07078 | mahuannin F | 1.06113 |
| Damascenone | 1.06111 | Gallocatechin- (4 → 6″; 2 → *O* → 7″)-(epi)gallocatechin | 1.03032 |
| (-)-*B*-Hydrastine | 1.0566 | Tetradecyldiethanolamine | 1.02484 |
| 2'-Hydroxy-*a*-naphthoflavone | 1.04948 | Methylophiopogonanone A | 1.02127 |
| Hexadecanoic acid | 1.04253 | ( +)-Catechin | 1.01432 |
| Cordycepin | 1.04157 | Sauchinone | 1.01409 |
| Schaftoside | 1.0343 | Oxypeucedanin hydrate | 1.01315 |
| Matairesinol | 1.01938 | Pseudoephedrine | 1.0123 |
| 5,7-Dihydroxy-3',4'-dimethoxyflavanone | 1.01709 | 1-Hexadecanoyl-sn-glycerol | 1.00627 |
| Benproperine | 1.01511 | Ephedradine B/D | 1.001 |
| Phytol | 1.00761 | 4-Ketozeinoxanthin | 1.00077 |
| Byakangelicin | 1.00415 |  |  |
| Erucylamide | 1.00325 |  |  |
